# Sex-related differences in prosthesis-patient mismatch after surgical aortic valve replacement and long-term outcomes

**DOI:** 10.1093/eurheartj/ehaf076

**Published:** 2025-02-18

**Authors:** Paolo Springhetti, Kathia Abdoun, Éric Dumont, François Dagenais, Dimitri Kalavrouziotis, Siamak Mohammadi, Philippe Pibarot, Marie-Annick Clavel

**Affiliations:** Institut Universitaire de Cardiologie et de Pneumologie, Université Laval, 2725 Chemin Ste-Foy, A-2047, Québec, QC, Canada G1V4G5; Institut Universitaire de Cardiologie et de Pneumologie, Université Laval, 2725 Chemin Ste-Foy, A-2047, Québec, QC, Canada G1V4G5; Institut Universitaire de Cardiologie et de Pneumologie, Université Laval, 2725 Chemin Ste-Foy, A-2047, Québec, QC, Canada G1V4G5; Institut Universitaire de Cardiologie et de Pneumologie, Université Laval, 2725 Chemin Ste-Foy, A-2047, Québec, QC, Canada G1V4G5; Institut Universitaire de Cardiologie et de Pneumologie, Université Laval, 2725 Chemin Ste-Foy, A-2047, Québec, QC, Canada G1V4G5; Institut Universitaire de Cardiologie et de Pneumologie, Université Laval, 2725 Chemin Ste-Foy, A-2047, Québec, QC, Canada G1V4G5; Institut Universitaire de Cardiologie et de Pneumologie, Université Laval, 2725 Chemin Ste-Foy, A-2047, Québec, QC, Canada G1V4G5; Institut Universitaire de Cardiologie et de Pneumologie, Université Laval, 2725 Chemin Ste-Foy, A-2047, Québec, QC, Canada G1V4G5

**Keywords:** Prosthesis-patient mismatch, Patient-prosthesis Mismatch, Sex differences, Aortic valve replacement

## Abstract

**Background and Aims:**

Prosthesis-patient mismatch (PPM) is associated with dismal prognosis after aortic valve replacement (AVR). Sex differences in PPM outcomes remain poorly explored. Therefore, this study aims to evaluate sex-specific impact in PPM after surgical AVR.

**Methods:**

Between 2000 and 2021, 7319 patients underwent surgical AVR at the Institut Universitaire de Cardiologie et de Pneumologie de Québec. Prosthesis-patient mismatch was defined by using the indexed effective orifice area (EOAi) and by applying the Valve Academic Research Consortium-3 (VARC-3) criteria. The cohort was followed up prospectively from surgical AVR until November 2023. The primary endpoint was defined as long-term mortality and the secondary endpoint as long-term cardiovascular (CV) and perioperative mortality. Mortality was established and CV mortality was adjudicated by Quebec national database.

**Results:**

Any-degree PPM resulted more prevalent in women than in men (31.9% vs. 19.7%, *P* < .0001) with rare incidence of severe PPM (2.4% vs. 0.6%, *P* < .0001) according to VARC-3 definition. Over a median follow-up of 12.6 years, there were 3231 (44.1%) all-cause deaths, with 1238 (16.9%) from CV causes. Prosthesis-patient mismatch was associated with all-cause mortality (hazard ratio 1.30, 95% CI 1.20–1.40; *P* < .0001) and CV mortality (hazard ratio 1.39, 95% CI 1.23–1.57; *P* < .0001) in the whole cohort without interaction between sexes (*P* ≥ .74). After comprehensive multivariable adjustment, VARC-3 PPM remained independently associated with outcome only in women (*P* ≤ .04). Adapting PPM definition according to spline-derived EOAi thresholds disaggregated by sex, PPM was independently associated with outcome in both sexes (*P* ≤ .04).

**Conclusions:**

Sex-specific EOAi thresholds associated with outcomes emerged in this large regional study. This finding suggests that PPM definition in men may follow higher EOAi thresholds than in women.

## Structured Graphical Abstract

**Structured Graphical Abstract:**
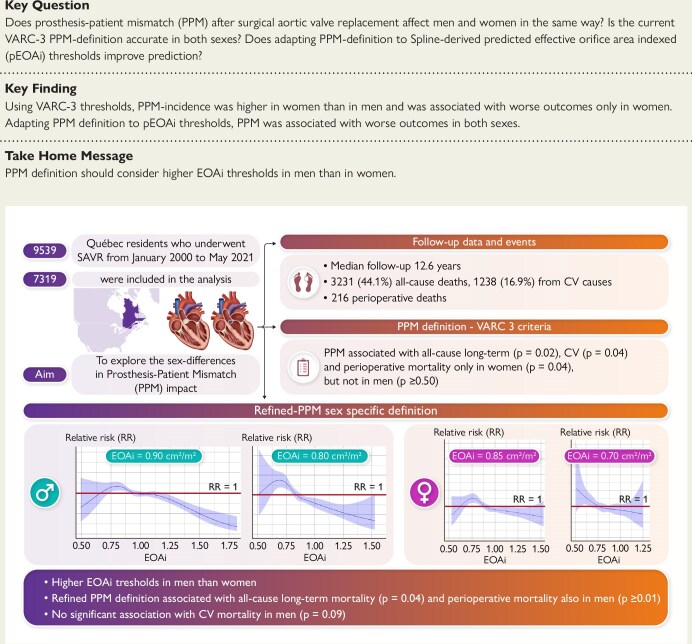
Sex differences in prosthesis-patient mismatch (PPM) after surgical aortic valve replacement. Men exhibited higher best indexed effective orifice area (EOAi) thresholds than women in predicting outcomes. Therefore, the Valve Academic Research Consortium-3 thresholds to define PPM were accurate in women, while in men the EOAi thresholds associated with impaired outcomes were higher than those proposed. Even after refining the PPM definition according to the EOAi sex-specific thresholds, women seemed to show higher PPM impact, driven mostly by cardiovascular mortality.

**See the editorial comment for this article ‘Patient-prosthesis mismatch after aortic valve replacement: one size does not fit all’, by F. Zito *et al*., https://doi.org/10.1093/eurheartj/ehaf200.**

## Introduction

Prosthesis-patient mismatch (PPM) after aortic valve replacement (AVR) occurs when the effective orifice area (EOA) of the implanted prosthesis is too small for the cardiovascular requirement of the patient.^1,2^ Prosthesis-patient mismatch is thus determined with the use of EOA indexed to body surface area (BSA) of the patient (EOAi). Recently, the Valve Academic Research Consortium-3 (VARC-3) proposed EOAi thresholds defining PPM severity with adjustment for patients with body mass index (BMI) ≥ 30 kg/m^2^ to avoid over-indexation of EOA and thus overestimation of PPM in obese patients.^3,4^ However, no previous research investigated specifically the best EOAi thresholds for PPM disaggregating by sex, leading to the same theoretical cut-off assumption in men and women.

Prosthesis-patient mismatch has been linked with worse short-^5^ and long-term^6–8^ cardiovascular outcomes, especially when PPM is severe,^8,9^ in younger patients^10,11^ and in patients with low ejection fraction or low flow.^5,12–14^ The preventive strategies to avoid PPM combined with technological improvement in the design of aortic valve prostheses and the surgical techniques^15^ as well as the rapidly growing utilization of transcatheter AVR,^16^ have contributed to reducing the incidence of PPM incidence in the past decade. Nevertheless, PPM still occurs after AVR, and its impact on outcomes remains important. Previous evidence suggests that PPM incidence is higher in women than in men and confirmed in different decades^10,17^; however, no study has evaluated the sex-specific impact of PPM after AVR.

Hence, this study sought to evaluate (i) the sex differences and temporal trends in the incidence of PPM, (ii) the short- and long-term mortality associated with PPM in a prospective cohort of patients who underwent AVR between 2000 and 2021, and (iii) the eventual association of EOAi as a continuous variable with long-term mortality and the identification of the best cut-off for PPM values disaggregated by sex.

## Methods

### Study design and population cohort

**Figure 1:**
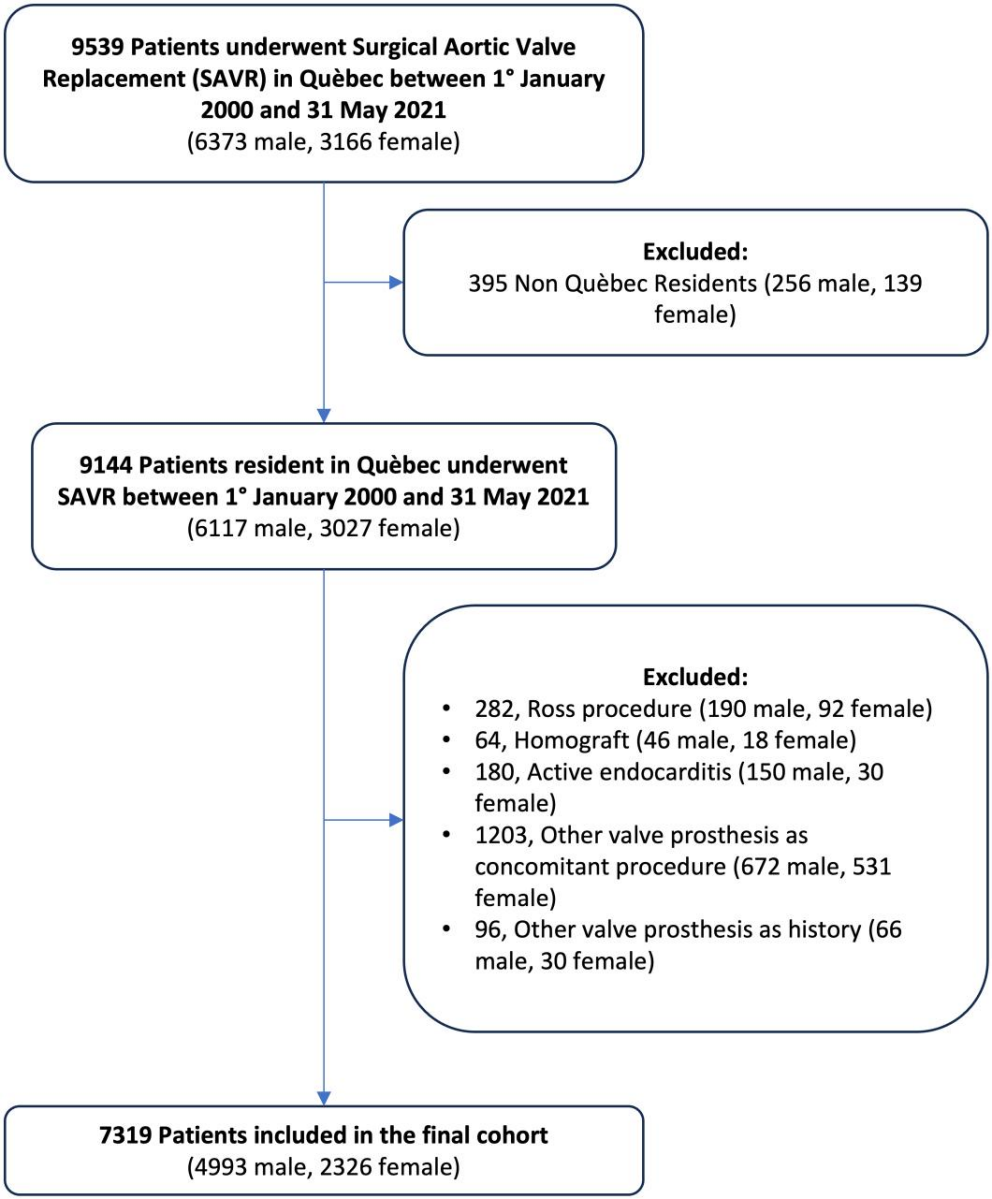
Flowchart of the study.

This was an observational, regional, population-based study. All consecutive adult patients who underwent surgical AVR for aortic stenosis at the Institut Universitaire de Cardiologie et de Pneumologie de Québec—Université Laval (Canada) between 1 January 2000 and 31 May 2021 were included. The exclusion criteria were non-Québec residents, Ross procedures, presence of aortic homograft, aortic dissection, previous intervention (surgical or transcatheter) on cardiac valves, or active endocarditis (*Figure 1*; *Structured Graphical Abstract*).

The study was approved by the research ethics committee of the Institut Universitaire de Cardiologie et de Pneumologie de Québec, and the participants gave written informed consent for the inclusion in the registry.

### Definition of prosthesis-patient mismatch

The normal reference values of EOAs for each given model and size of prosthetic valve were obtained from the literature (see Supplementary data online, *Table S1*) and indexed to patient’s BSA (EOAi), obtained with the Mosteller method.^18^ According to VARC-3 criteria,^3^ absence of PPM was defined independently of sex as EOAi >0.85 cm^2^/m^2^ in patients with BMI <30 kg/m^2^ or EOAi >0.70 cm^2^/m^2^ in patients with BMI ≥30 kg/m^2^, moderate PPM as EOAi 0.85–0.66 cm^2^/m^2^ (BMI <30 kg/m^2^) or EOAi >0.70–0.56 cm^2^/m^2^ (BMI ≥30 kg/m^2^), and severe PPM as EOAi ≤0.65 cm^2^/m^2^ in patients with BMI <30 kg/m^2^ or EOAi ≤0.55 cm^2^/m^2^ in patients with BMI ≥30 kg/m^2^.

### Outcomes

The primary outcome was all-cause mortality. The secondary outcomes were (ⅰ) mortalities only determined by underlying cardiovascular causes and (ⅱ) operative mortality (i.e. during the same hospitalization subsequent to the operation or within 30 days). All deaths were adjudicated and collected by the ‘Institut de la statistique de Québec’ and included in the registry prospectively every 6 months. To maximize the interrogation of this provincial database, a list with multiple demographics (including first and last names, dates of birth, and social security numbers) and a delay of 6 months between the last interrogation and closing follow-up dates were used. The end of the follow-up was 28 November 2023, and the follow-up was 100% complete. The adjudication of the underlying cause of death (i.e. cardiovascular) was standardized in the registry starting from 2015, according to published recommendations,^19^ and further updated in 2018.^20^

### Statistical methods

Continuous variables were presented as mean ± SD or median with interquartile range if not normally distributed. The differences between PPM severity groups were assessed using Student’s *t*-test or Wilcoxon–Mann–Whitney test as appropriate. Categorical variables were expressed as absolute numbers and percentages and compared with χ^2^ test. The Kaplan–Meier method was used to assess the survival between PPM groups. Then, uni- and multivariable Cox proportional hazards models were used to evaluate PPM as an independent predictor of all-cause and cardiovascular mortality. Results are presented as adjusted hazard ratios (aHR) with 95% confidence intervals. The Cox models were adjusted for clinically relevant variables and those found to be significant in univariable analysis with the outcome, after ensuring the exclusion of collinearity (displayed by correlation matrix—Supplementary data online, *Figure S1*). Univariable and multivariable logistic regression analyses were conducted to evaluate the impact of PPM on 30-day mortality. Adjustments in the multivariable models were made using only the European System for Cardiac Operative Risk Evaluation II (EuroSCORE II),^21^ to avoid overfitting. Results are presented as adjusted odds ratio (aOR) with 95% confidence intervals.

Moreover, cubic splines with three knots disaggregated by sex and BMI were computed to assess the association between EOAi as a continuous variable and the relative risk (RR) for long-term mortality. The spline curve knots’ number was established by the optimization of the Akaike information criterion (AIC). The best cutoffs were defined whereby an excessive mortality threshold (intersection with RR = 1)^22–24^ was identified in the cohort, separately in men and women.

Patients with EOAi values below the derived EOAi thresholds were further classified in the PPM group, according to sex and BMI. This refined PPM definition was updated in uni- and multivariable Cox proportional hazards models and logistic regression analysis for predicting long-term/cardiovascular mortality and perioperative mortality, respectively. Hierarchical χ^2^ models have been additionally computed to investigate if the addition of the refined PPM definition improves the baseline model including several clinical and demographical covariates. To ensure the predictive power of the Cox models, time receiver operating characteristic curves were computed, assessing the difference with DeLong test. Akaike information criterion and the calibration plots adopting the Hosmer–Lemeshow test were additionally computed for the multivariable models.

Finally, the effect of age on the association between PPM and survival was explored through Kaplan–Meier estimator and normogram was designed to predict the 10-year survival after AVR from the multivariable analysis of all-cause death for different ages in relation to EOAi as a continuous variable.

Statistical analyses were carried out with JMP 16.2 (SAS Institute Inc., Cary, NC, USA) and R Studio version 4.3.2. A *P*-value <.05 was considered statistically significant.

## Results

### Population and prosthesis-patient mismatch incidence

In the final analysis, we included 7319 patients who underwent AVR between 2000 and 2021, after applying exclusion criteria. The mean age of the population was 69 ± 10 years and 2326 (32%) patients were female. Overall, the incidence of any-degree PPM mismatch was 22.8%, more frequent in female than in male patients (31.9% vs. 19.7%, *P* < .0001). Sex-specific baseline characteristics are presented in *Table 1*.

**Table 1:** Baseline characteristics in the whole cohort and according to sex

	Overall (*n* = 7319)	Male (*n* = 4,993, 68.2%)	Female (*n* = 2,326, 31.8%)	*P*-value^a^
Age (years)	69.2 ± 10.2	68.1 ± 10.3	71.5 ± 9.8	**<.0001**
BSA (m^2^)	1.89 ± 0.23	1.97 ± 0.21	1.72 ± 0.20	**<.0001**
BMI (kg/m^2^)	28.3 ± 5.3	28.2 ± 5.0	28.4 ± 5.9	.21
Diabetes	1989 (27.2%)	1368 (27.4%)	621 (26.7%)	.53
Hypertension	5293 (72.3%)	3557 (71.2%)	1736 (74.6%)	.**002**
Dyslipidemia	5908 (80.7%)	4074 (81.6%)	1834 (78.8%)	.**006**
Atrial fibrillation	1007 (13.8%)	688 (13.8%)	319 (14.7%)	.94
Coronary artery disease	3643 (49.8%)	2776 (55.6%)	867 (37.3%)	**<.0001**
Myocardial infarction	1300 (17.8%)	1003 (20.1%)	297 (12.8%)	**<.0001**
COPD	836 (11.4%)	571 (11.4%)	265 (11.4%)	.96
Smoking status	837 (11.4%)	602 (12.6%)	235 (10.1%)	.**01**
Previous stroke or TIA	671 (9.2%)	464 (9.3%)	207 (8.9%)	.59
CKD	241 (3.3%)	197 (3.9%)	33 (1.9%)	**<.0001**
eGFR (mL/min/1.73 m^2^)	68.9 ± 24.6	73.8 ± 24.6	58.4 ± 21.1	**<.0001**
NYHA Class III or IV	2859 (39.1%)	1747 (35.9%)	1112 (47.8%)	**<.0001**
Angina (CCS Class 4 or 5)	1258 (17.2%)	931 (18.6%)	327 (14.1%)	**<.0001**
EuroSCORE II	2.8 (1.6–5.1)	2.6 (1.4–4.7)	3.3 (1.8–5.8)	**<.0001**
**Echocardiographic data**				
LVEF (%)	57.9 ± 11	56.8 ± 11.7	60.4 ± 9.8	**<.0001**
Left atrium (mL/m^2^)	40.3 ± 7.2	41.1 ± 7.4	38.8 ± 6.6	**<.0001**
LVEDd (mm)	48.2 ± 7.6	49.8 ± 7.5	44.5 ± 6.5	**<.0001**
LVMi (g/m^2^)	110.0 ± 36.6	114.8 ± 37.8	99.1 ± 32.0	**<.0001**
RWT	0.48 ± 0.13	0.47 ± 0.12	0.50 ± 0.16	**<.0001**
Max gradient (mmHg)	66.7 ± 29.1	65.1 ± 29.2	70.3 ± 28.7	**<.0001**
Mean gradient (mmHg)	40.4 ± 18.4	39.3 ± 18.3	42.7 ± 18.6	**<.0001**
**Operative data**				
Valve size (mm)	23.5 ± 2.3	24.4 ± 2.0	21.6 ± 1.7	**<.0001**
Mechanical prosthesis	891 (12.1%)	648 (13.0%)	243 (10.4%)	.**002**
Predicted EOAi (cm^2^/m^2^)	0.91 ± 0.17	0.93 ± 0.17	0.88 ± 0.16	**<.0001**
PPM (any grade)	1667 (22.8%)	926 (19.7%)	741 (31.9%)	**<.0001**
PPM moderate	1583 (21.6%)	898 (18.0%)	685 (29.4%)	**0.0001**
PPM severe	84 (1.15%)	28 (0.56%)	56 (2.4%)	**<.0001**

Data are expressed as mean ± SD or median (interquartile range).

Bold values represent statistically significant differences (i.e. *P* < .05).

BMI, body mass index; BSA, body surface area (Mosteller method); CCS, Canadian Cardiovascular Society; NYHA, New York Heart Association; COPD, chronic obstructive pulmonary disease; CAD, coronary artery disease; CKD, chronic kidney disease; eGFR, estimated glomerular filtration rate (Cockcroft–Gault formula); LVEDd, left ventricular end-diastolic diameter; LVEF, left ventricular ejection fraction; LVMi, left ventricular mass index; PPM, prosthesis-patient mismatch; RWT, relative wall thickness; TIA, transient ischaemic attack.

^a^ Obtained with t-test or Wilcoxon–Mann–Whitney test for continuous variables as appropriate, or χ2 for categorical variables.

On average, patients with any degree of PPM were older, with smaller left ventricular geometry, higher left ventricular ejection fraction, and higher pre-operative transvalvular peak/mean gradients (*Table 2*). Severe PPM was observed only in 84 patients (1.15%) of the whole cohort, more frequent in females than in males (2.4% vs. 0.6%, *P* < .0001). There was a temporal trend of decrease in PPM from 2000 to 2021, in both males (*P* < .0001) and females (*P* = .014) (see Supplementary data online, *Figures S2* and *S3*). Nevertheless, without significant differences in the slopes’ decline over time at linear regression analysis disaggregating by sex (*Z*-value 0.21, *P* = .83) (see Supplementary data online, *Figure S4*).

**Table 2:** Baseline characteristics of patients according to sex and prosthesis-patient mismatch

	Male *n* = 4993			Female *n* = 2326		
	No PPM (*n* = 4067; 81.5%)	PPM (*n* = 926; 18.5%)	*P*-value^a^	No PPM (*n* = 1585; 68.1%)	PPM (*n* = 741; 31.9%)	*P*-value^a^
Age (years)	67.8 ± 10.4	69.8 ± 9.8	**<.0001**	71.2 ± 9.6	72.4 ± 9.8	.**006**
BSA (m^2^)	1.96 ± 0.21	1.97 ± 0.19	.21	1.75 ± 0.21	1.73 ± 0.19	.29
BMI (kg/m^2^)	28.3 ± 5.1	27.8 ± 4.3	.**002**	28.4 ± 6.2	28.3 ± 5.4	.54
Diabetes	1100 (27.8%)	268 (28.9%)	.24	390 (24.6%)	231 (31.2%)	.**0009**
Hypertension	2885 (71.2%)	672 (72.5%)	.32	1171 (73.4%)	565 (76.2%)	.22
Dyslipidemia	3304 (81.4%)	770 (83.1%)	.17	1229 (77.5%)	605 (81.6%)	.**02**
Atrial fibrillation	567 (13.9%)	121 (13.1%)	.48	214 (13.5%)	105 (14.2%)	.66
Coronary artery disease	2232 (54.9%)	544 (58.8%)	.**03**	553 (34.9%)	314 (42.4%)	.**0005**
Previous myocardial infarction	803 (19.7%)	200 (21.6%)	.21	205 (12.9%)	92 (12.4%)	.73
COPD	452 (11.1%)	119 (12.8%)	.14	188 (11.9%)	77 (10.4%)	.29
Smoking status	3575 (87.9)	816 (88.1)	.85	156 (9.8%)	79 (10.7%)	.54
Previous stroke or TIA	359 (8.8%)	105 (11.3%)	.**02**	148 (9.3%)	59 (8.0%)	.27
CKD	154 (3.8%)	43 (4.6%)	.24	30 (1.9%)	14 (1.9%)	.99
eGFR (mL/min/1.73 m^2^)	73.9 ± 24.4	73.2 ± 25.0	.42	58.8 ± 21.5	57.5 ± 20.3	.14
NYHA Class III or IV	1412 (34.7%)	335 (36.2%)	.40	728 (45.9%)	384 (51.8%)	.**008**
Angina (CCS Class 4 or 5)	763 (18.8%)	168 (18.1%)	.66	224 (14.1%)	103 (13.9%)	.88
EuroSCORE II	2.6 (1.4–4.7)	2.6 (1.4–4.8)	.72	3.2 (1.8–5.6)	3.4 (1.9–6.2)	.14
**Echocardiographic data**						
LVEF (%)	56.6 ± 11.7	57.7 ± 11.4	.**015**	60.0 ± 9.7	61.2 ± 9.9	.**01**
Left atrium index (mL/m^2^)	41.1 ± 7.4	41.3 ± 7.0	.76	38.5 ± 6.5	39.2 ± 6.8	.**15**
LVEDd (mm)	50.2 ± 7.7	48.3 ± 6.7	**<.0001**	44.7 ± 6.8	44.1 ± 5.8	.**04**
LVMi (g/m^2^)	116.5 ± 39.0	107.3 ± 33.5	**<.0001**	100.7 ± 33.6	95.8 ± 27.8	.**003**
RWT	0.47 ± 0.12	0.49 ± 0.12	.**006**	0.50 ± 0.26	0.49 ± 0.12	.**47**
Peak gradient (mmHg)	64.4 ± 29.4	68.7 ± 28.0	**<.001**	70.0 ± 30.0	70.9 ± 25.7	.**51**
Mean gradient (mmHg)	38.9 ± 18.4	41.1 ± 18.0	.**0003**	42.7 ± 19.2	42.8 ± 19.8	.**88**
**Operative data**						
Valve size (mm)	24.7 ± 0.03	23.1 ± 0.06	**<.0001**	22.1 ± 1.6	20.4 ± 1.3	**<.0001**
Mechanical prosthesis	538 (13.2%)	110 (17.0%)	.27	158 (10.0%)	85 (11.5%)	.27

Data are expressed as mean ± SD or median (interquartile range).

Bold values represent statistically significant differences (i.e. *P* < .05).

BMI, body mass index; BSA, body surface area (Mosteller method); CCS, Canadian Cardiovascular Society; NYHA, New York Heart Association; COPD, chronic obstructive pulmonary disease; CAD, coronary artery disease; CKD, chronic kidney disease; eGFR, estimated glomerular filtration rate (Cockcroft–Gault formula); LVEDd, Left ventricular end-diastolic diameter; LVEF, left ventricular ejection fraction; LVMi, left ventricular mass index; RWT, relative wall thickness; TIA, transient ischaemic attack.

^a^Obtained with *t*-test or Wilcoxon–Mann–Whitney test for continuous variables as appropriate, or χ^2^ for categorical variables.

### Survival analysis according to Valve Academic Research Consortium-3 criteria

**Figure 2:**
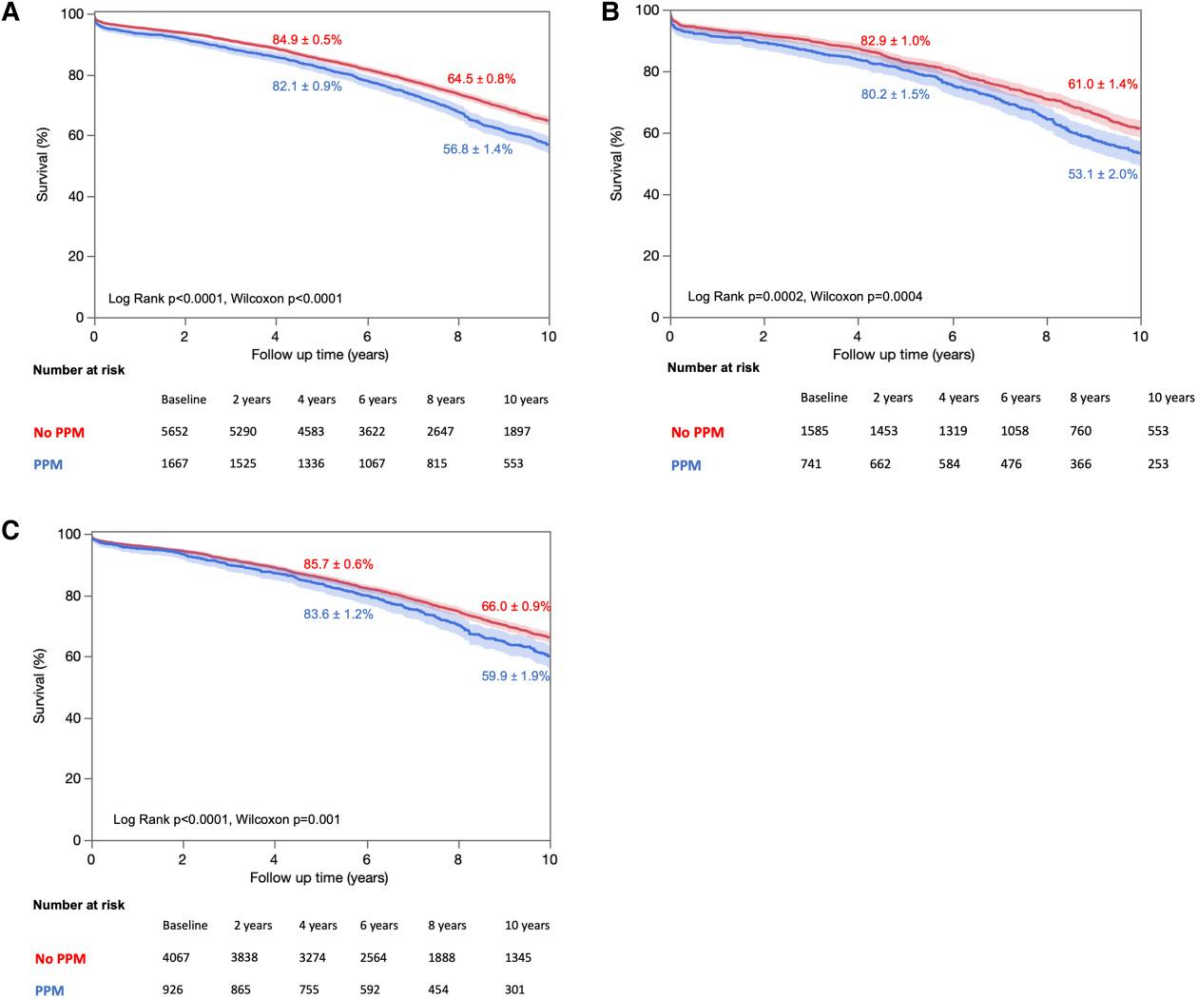
Kaplan–Meier curves of survival according to presence or absence of prosthesis-patient mismatch. Overall survival in (*A*) the whole cohort, (*B*) female patients, and (*C*) male patients. Event rates are expressed ad mean ± SD. CV, cardiovascular; PPM, prosthesis-patient mismatch.

**Figure 3:**
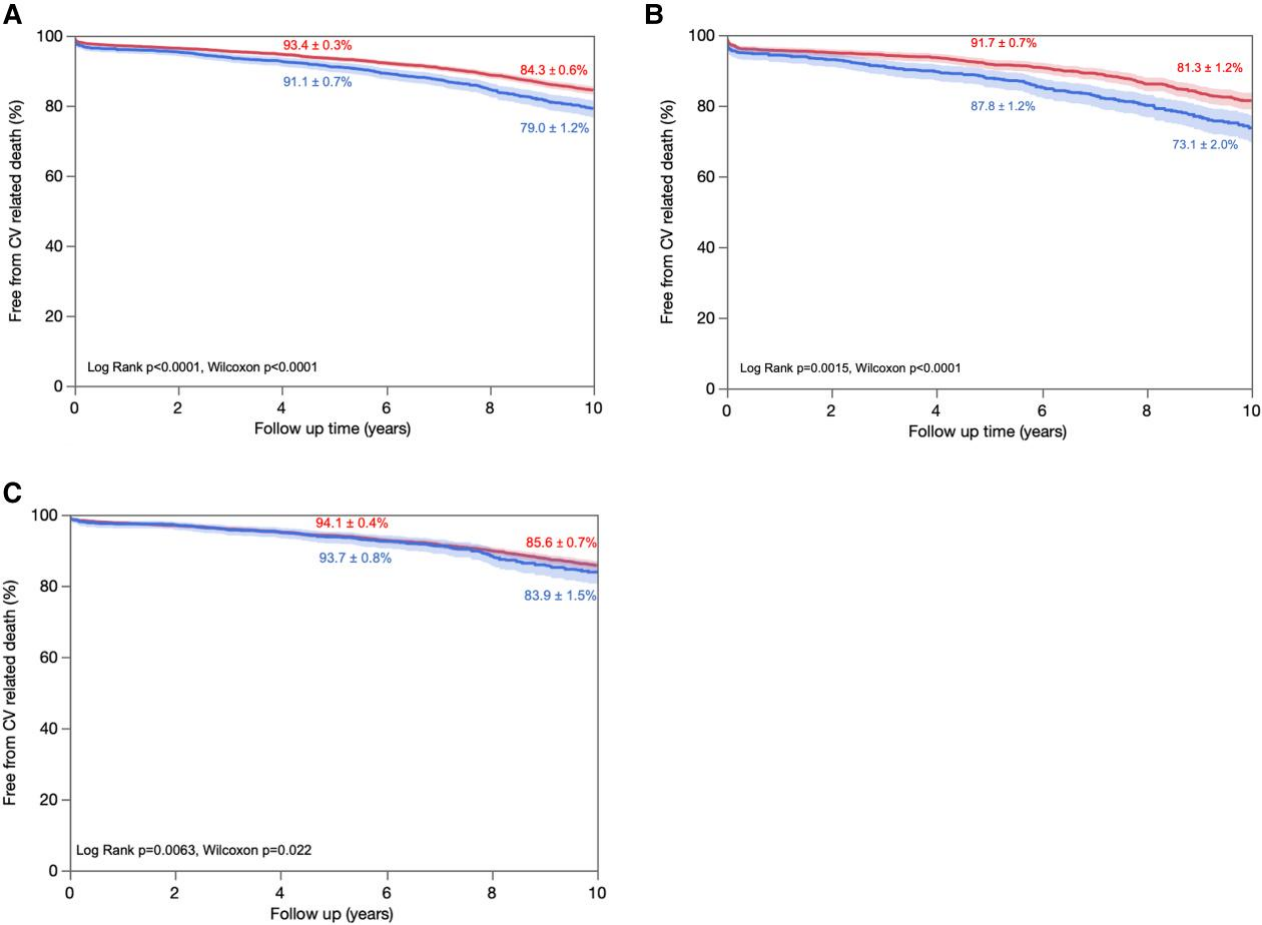
Kaplan–Meier curves of cardiovascular survival according to presence or absence of prosthesis-patient mismatch. Overall survival in (*A*) the whole cohort, (*B*) female patients, and (*C*) male patients. Event rates are expressed ad mean ± SD. CV, cardiovascular; PPM, prosthesis-patient mismatch.

The median follow-up of the cohort was 12.6 (interquartile range 12.3–12.9) years, during which 3231 (44.1%) all-cause deaths occurred and 1238 (16.9%) from cardiovascular causes. Patients with PPM presented a 30% increase in mortality compared with patients without PPM [hazard ratio (HR) 1.30, 95% CI 1.20–1.40; *P* < .0001], with a survival rate at 5 years of 82 ± 1% vs. 85 ± 1% and at 10 years of 57 ± 1% and 65 ± 1%, respectively (*P* < .0001) (*Figure 2A*). Interestingly, there was no interaction between sex and PPM regarding survival (univariate: *P* = .94; multivariate: *P* = .33), with univariate HR 1.25 (95% CI 1.13–1.40; *P* < .0001) in female patients and HR 1.25 (95% CI 1.12–1.41; *P* = .0002) in male patients. Kaplan–Meier curves disaggregated by sex are represented in *Figure 2B* and *C*, respectively, for women and men. Similarly, patients with PPM had a higher cardiovascular mortality rate than patients without PPM (HR 1.39, 95% CI 1.23–1.57; *P* < .0001), which was not different (*P* = .74) between female (HR 1.33, 95% CI 1.12–1.56; *P* = .002) and male (HR 1.28, 95% CI 1.07–1.52; *P* = .006) patients (*Figure 3*).

After comprehensive adjustment for age, previous transient ischaemic attack (TIA)/stroke, hypertension, diabetes, chronic obstructive pulmonary disease, coronary artery disease, chronic kidney disease (CKD), atrial fibrillation, smoking, New York Heart Association (NYHA) class, left ventricular ejection fraction, type of prosthesis (biological vs. mechanical), PPM remained independently associated with all-causes and cardiovascular mortality in female patients (aHR 1.18, 95% CI 1.02–1.36; *P* = .02 and aHR 1.21, 95% CI 1.01–1.46; *P* = .04, respectively; *Table 3* and Supplementary data online, *Table S2*), but not in male patients (aHR 1.02, 95% CI 0.90–1.16; *P* = .69 and aHR 1.05, 95% CI 0.87–1.27; *P* = .66, respectively; *Table 4* and Supplementary data online, *Table S3*). The calibration plot adopting Hosmer–Lemeshow test is reported in Supplementary data online, *Figure S5*. Time receiver operating characteristic (ROC) area under the curve (AUC) at 15 years for all-cause mortality in this model is 0.826 and 0.837, respectively, for women and men (see Supplementary data online, *Figure S6*). In addition, PPM added incremental value for predicting long-term mortality in women but not in men (likelihood ratio test 4.36, *P* = .035 and .80, *P* = .41, respectively).

**Table 3:** Multivariable Cox-regression of overall long-term mortality in women (prosthesis-patient mismatch defined by Valve Academic Research Consortium-3 definition)

	Model 1 VARC-3 definition^a^	
	HR (95% CI)	*P*-value
Age (1 year increase)	1.07 (1.06–1.09)	**<.0001**
BMI (1 unit increase)	1.01 (0.99–1.02)	.11
Atrial fibrillation	1.61 (1.33–1.94)	**<.0001**
Previous stroke or TIA	1.2 (0.95–1.50)	.11
Diabetes	1.48 (1.27–1.73)	**<.0001**
Hypertension	1.02 (0.86–1.22)	.81
Dyslipidemia	1.30 (1.10–1.54)	.**003**
COPD	1.45 (1.19–1.77)	.**0002**
CAD	1.19 (1.02–1.39)	.**03**
CKD	2.04 (1.37–3.05)	.**0005**
Smoking status	1.86 (1.44–2.38)	**<.0001**
NYHA III/IV	1.14 (0.99–1.31)	.06
Angina CCS 4/5	1.03 (0.83–1.27)	.79
LVEF (1% increase)	0.98 (0.97–0.99)	.**03**
Mechanical prosthesis	1.19 (0.90–1.57)	.21
**PPM**	1.18 (1.02–1.36)	.**02**

Bold values represent statistical significance (i.e. *P* < .05).

BMI, body mass index; CCS, Canadian Cardiovascular Society angina grading; NYHA, New York Heart Association; COPD, chronic obstructive pulmonary disease; CAD, coronary artery disease; CKD, Chronic Kidney Disease; LVEF, left ventricle ejection fraction; TIA, transient ischaemic attack; PPM, Prosthesis-patient mismatch.

^a^VARC 3 definition-Effective Orifice Area Index thresholds align to those derived by Spline Curve Analysis, thus the model remains the same.

**Table 4:** Multivariable Cox-regression of long-term mortality in men (prosthesis-patient mismatch defined by Valve Academic Research Consortium-3 definition vs. spline curve thresholds)

	Model 1 VARC-3 definition		Model 2 Spline curve thresolds^a^	
	HR (95% CI)	*P*-value	HR (95% CI)	*P*-value
Age (1 year increase)	1.26 (1.07–1.49)	**<.0001**	1.08 (1.07–1.10)	**<.0001**
BMI (1 unit increase)	1.0 (0.99–1.02)	.48	1.00 (0.99–1.01)	0.52
Atrial fibrillation	1.23 (1.05–1.39)	.**007**	1.19 (1.05–1.35)	.**0049**
Previous stroke or TIA	1.26 (1.07–1.49)	.**006**	1.27 (1.10–1.45)	.**0007**
Diabetes	1.46 (1.30–1.64)	**<.0001**	1.43 (1.29–1.57)	**<.0001**
Hypertension	1.08 (0.97–1.22)	.16	1.05 (0.95–1.16)	.36
Dyslipidemia	1.25 (1.09–1.43)	.**0012**	1.26 (1.12–1.41)	**<.0001**
COPD	1.69 (1.47–1.94)	**<.0001**	1.80 (1.60–2.02)	**<.0001**
CAD	1.27 (1.13–1.43)	**<.0001**	1.37 (1.24–1.51)	**<.0001**
CKD	2.06 (1.69–2.50)	**<.0001**	2.16 (1.83–2.56)	**<.0001**
Smoking status	1.51 (1.29–1.77)	**<.0001**	1.49 (1.31–1.71)	**<.0001**
NYHA III/IV	1.13 (1.02–1.26)	.**02**	1.14 (1.04–1.25)	.**007**
Angina CCS 4/5	1.01 (088–1.17)	.82	1.02 (0.90–1.15)	.74
LVEF (1% increase)	0.98 (0.98–0.99)	**<.0001**	0.99 (0.98–0.99)	**<.0001**
Mechanical prosthesis	1.30 (1.06–1.59)	.**01**	1.20 (1.01–1.42)	.**04**
**PPM**	1.02 (0.90–1.16)	.69	1.10 (1.01–1.20)	.**04**

Bold values represent statistical significance (i.e. *P* < .05).

BMI, body mass index; CCS, Canadian Cardiovascular Society angina grading; NYHA, New York Heart Association; COPD, chronic obstructive pulmonary disease; CAD, coronary artery disease; CKD, Chronic Kidney Disease; LVEF, left ventricle ejection fraction; TIA, transient ischaemic attack; PPM, Prosthesis-patient mismatch.

^a^Effective Orifice Area Indexed (EOAi) < 0.90 if BMI <30 kg/m^2^, EOAi < 0.80 if BMI ≥ 30 kg/m^2^.

There were 216 (2.95%) operative deaths, 107 (4.60%) in female patients and 109 (2.18%) in male patients. Prosthesis-patient mismatch was associated with operative mortality in the whole cohort (OR 1.55, 95% CI 1.15–2.07; *P* = .004), without interaction between sexes (*P* = .25). However, PPM was associated with operative mortality in female patients (OR 1.58, 95% CI 1.07–2.36; *P* = .02) but not in male patients (OR 1.11, 95% CI 0.69–1.88; *P* = .66). After adjustment by EuroSCORE II, PPM remained associated with increased operative mortality in female patients only (female: aOR 1.52, 95% CI 1.01–2.30; *P* = .04; male: aOR 1.19, 95% CI 0.72–1.95; *P* = .50). Supplementary data online, *Tables S4* and *S5* are detailing the logistic regression models, respectively, in women and men.

### Refining prosthesis-patient mismatch definition according to sex with spline-derived indexed effective orifice area thresholds

**Figure 4:**
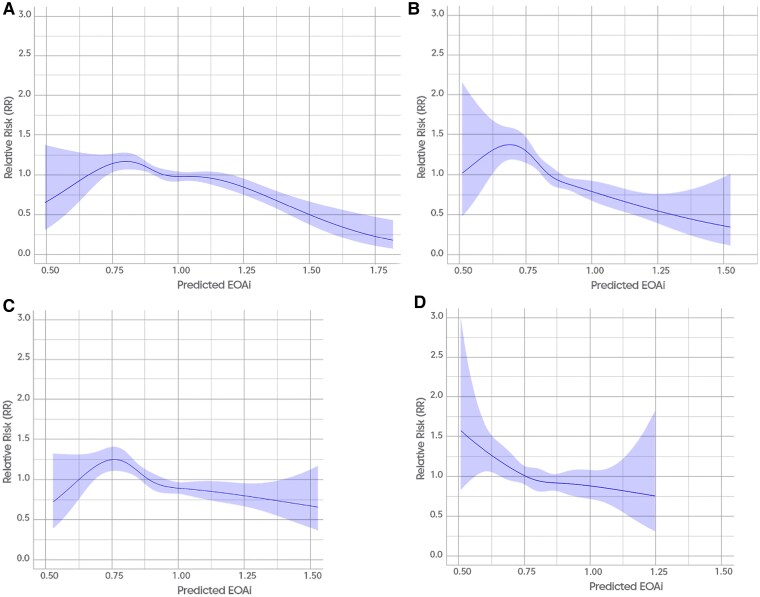
Spline curve analysis of predicted indexed effective orifice area (continuous variable) and long-term mortality. Long-term mortality associated with indexed effective orifice area in (*A*) men with body mass index <30 kg/m^2^, (*B*) men with body mass index ≥30 kg/m^2^, (*C*) women with body mass index <30 kg/m^2^, and (*D*) women with body mass index ≥30 kg/m^2^. RR is shown on the *y*-axis, while the indexed effective orifice area as continuous variable is reported on the *x*-axis. 95% confidence intervals are displayed in all the graphs. BMI, body mass index; EOAi, indexed effective orifice area.

The risk of all-cause death according to EOAi (continuous variable) is displayed by spline curves, according to sex and BMI (*Figure 4*). Notably, different EOAi thresholds are derived after stratification for sex and BMI. The best EOAi cut-off for men with BMI <30 kg/m^2^ is around 0.90 cm^2^/m^2^ (*Figure 4A*), lower compared with women with BMI <30 kg/m^2^ (around 0.85 cm^2^/m^2^; *Figure 4C*). On the contrary, as established by VARC-3 definition, the EOAi thresholds in patients with BMI ≥30 kg/m^2^ were lower compared with those with BMI <30 kg/m^2^ (around 0.80 and 0.70 cm^2^/m^2^, for men and women, respectively; *Figure 4B* and *D*; *Structured Graphical Abstract*).

Applying those cut-offs in a refined PPM definition, 1630 men (32.6%) are classified as having any-degree PPM, which was comparable to women (31.9%; *P* = .50).

Cox multivariable analyses for long-term all-cause and cardiovascular mortality were further conducted after refining the PPM definition with spline-derived cut-offs; the time ROC curve at 15 years for all-cause mortality shows a significant increase in the AUC substituting the refined PPM to the VARC-3 definition in the model for men (AUC 0.837 vs. AUC 0.840, DeLong test *P* = .027) (see Supplementary data online, *Figure S4B*). AIC provided similar results.

The refined PPM definition emerged in men as an independent predictor of long-term all-cause mortality after comprehensive multivariable adjustment (aHR 1.10, 95% CI 1.01–1.20, *P* = .04; *Table 4*) but not for cardiovascular mortality (aHR 1.14, 95% CI 0.98–1.33, *P* = .09; Supplementary data online, *Table S3*). Furthermore, the inclusion in men of the refined PPM definition in a hierarchical model significantly improves the χ^2^ for long-term all-cause mortality on top of the covariates (incremental χ^2^ 4.0, *P* = .04), but not for cardiovascular mortality (incremental χ^2^ 2.81, *P* = .09).

Finally, refined PPM definition presented a significant association with operative mortality in men (OR 1.52, 95% CI 1.03–2.23; *P* = .03). After adjustment by EuroSCORE II, PPM remained associated with increased operative in male (male: aOR 1.67, 95% CI 1.11–2.53; *P* = .01; Supplementary data online, *Table S5*).

### Effect of age on prosthesis-patient mismatch impact

At baseline, elderly men with PPM (≥70 years) exhibited a higher comorbidity burden than elderly women, as dyslipidaemia, previous stroke or TIA, CKD, and advanced NYHA class were more prevalent only in elderly males (see Supplementary data online, *Table S6*). Disaggregating by sex and age at survival analyses, younger men and elderly women with PPM showed higher all-cause death rates than younger men and elderly women without PPM, respectively (see Supplementary data online, *Figure S7*).

After adjustment, there was no effect of age on the relationship between PPM and survival impairment. The normogram in Supplementary data online, *Figure S8* displays the predicted 10-year survival after AVR from the multivariable analysis of death for different ages in relation to EOAi as a continuous variable. As the figure shows, the reduction of EOAi resulted in a similar 10-year survival decrease in different ages in both sexes (similar shape of the different curves).

## Discussion

The main findings from our investigation can be summarized as follows: (i) the incidence of PPM decreased over the past two decades, particularly in men and the overall incidence was 22.8% for PPM of any degree and only 1.15% for severe PPM; (ii) the incidence of PPM is significantly (*P* < .0001) higher in women compared with men, with rates for any-degree PPM being 31.9% vs. 19.7%, and for severe PPM, 2.4% vs. 0.6%, respectively—according to the current VARC-3 definition; (iii) the EOAi thresholds associated with long-term mortality resulted different in men and women, suggesting that the current PPM definition may be refined accounting not only for BMI but also for sex; (iv) in both women and men, individuals diagnosed with PPM experience worse long-term all-cause and cardiovascular mortality, with more impact in women in cardiovascular long-term mortality even after applying the refined PPM definition; and (v) the independent association between PPM and perioperative mortality is observed in both sexes, with a ∼1.5-fold increase in mortality, in both men and women after adopting the refined PPM definition (*Structured Graphical Abstract*).

### Incidence of prosthesis-patient mismatch: temporal trends and definition

This study revealed an incidence of any-degree PPM lower than in most previous studies, but similar to the evaluation of the surgical AVR arm of the PARTNER trials.^9^ Specifically, 22.8% of patients presented any-degree PPM and 1.15% of severe PPM. In order to compare the incidence of PPM between the different studies, the method to measure and the definition of PPM must be closely considered. Indeed, many studies used the post-AVR measure of EOA to calculate PPM. However, given the presence of low flow status, the underestimation of EOA post-AVR is frequent, and thus the incidence of PPM is overestimated.^25,26^ Moreover, the use of the new VARC-3 definition of PPM, with the decreased thresholds of EOAi in obese patients also decrease the incidence of PPM post-AVR.^3^ Finally, the haemodynamic improvement of surgical prosthetic valves over the past decades contributed to reducing PPM.

The evolution of PPM thresholds over time warrants attention. The original PPM definition did not rely on BMI differences, subsequently introduced by VARC-3 criteria.^3^ This inclusion represents the pivotal advancement towards the optimization of the PPM cut-offs considering individual baseline characteristics. The present study underscores and strengthens this trend towards personalized medicine, showing that important sex-related differences in terms of cut-offs may be additionally present in PPM definition. Our findings indicate that EOAi thresholds for women are consistent with the VARC-3 definition, while men may require higher cut-offs. This study is the first one to demonstrate in a large cohort sex-specific EOAi thresholds linked to adverse outcomes.

This discrepancy likely stems from inherent sex differences in cardiovascular demand for similar BSA values, with men generally necessitating a higher cardiovascular output^23^ and the smaller heart cavities and vessels in women (after adjustment for BSA). Therefore, the rationale for a higher EOAi threshold in men may be attributed to these physiological differences.

### Impact of prosthesis-patient mismatch on perioperative mortality

Prosthesis-patient mismatch was associated with perioperative mortality in the whole cohort, as previously reported.^27^ However, in the present study, this association was driven by female patients, as there was no association between PPM and operative mortality in male patients, despite the same number of events in both sexes, adopting not sex-corrected cut-offs. Interestingly, previous studies that found an association between PPM and mortality were the ones with low flow or low left ventricular ejection fraction patients or with a larger number of women.^5,28,29^ Strikingly, the independent PPM outcome link was found after applying sex-specific EOAi thresholds also in men. Concomitant bypass surgery and the baseline comorbidity burden may have also played a role in the perioperative mortality determination.

### Impact of prosthesis-patient mismatch on long-term mortality

In the whole cohort, patients with PPM present worse long-term outcome. After multivariable adjustment, PPM remained an independent predictor of outcome, accounting for a 1.2-fold increased risk of all-cause mortality, which is concordant with previous studies and meta-analysis.^6,8,17,30^ However, despite no obvious interaction between sex and PPM with regard to increased mortality, PPM remained an independent predictor of mortality in women but not in men.

We already demonstrated in our meta-analysis that the effect of PPM was modulated by female sex, with a stronger association between PPM and mortality in populations with larger proportion of women.^8^ This finding was obtained using the current VARC-3 criteria, where the proposed EOAi thresholds for PPM definition align with those derived from spline curve analysis only in women, but not in men. Notably, when PPM was refined according to sex-specific EOAi thresholds, it emerged as an independent predictor of mortality in men as well, reinforcing the need to shift the cut-off to higher values in male patients.

In survival analyses, younger men and older women with PPM showed higher all-cause death rates than their counterparts without PPM. Elderly men exhibit a higher comorbidity burden than women, with dyslipidaemia, previous stroke/TIA, CKD, and advanced NYHA class more prevalent in men. This may weaken the association between PPM and outcomes in older men, whereas it remains evident in women at survival analysis due to their lower comorbidity burden. In younger men, PPM impact remains greater, likely due to their higher activity levels and longer life expectancy with PPM effect exposure.

The lack of an observed age effect on the relationship between PPM and survival impairment in the normogram, accounting for comprehensive baseline adjustment, contrasts with previous evidence from the pre-transcatheter AVR era, which used different methodological approaches (geometrical orifice area).^31^ However, Dismorr *et al*.^17^ recently found that the interaction of age when added to PPM resulted not significant in predicting compound outcome effects, aligning with our finding.

### Impact of prosthesis-patient mismatch in women: a pathophysiological perspective

Aortic stenosis is a pathological condition that encompasses both the valve and the left ventricle (LV), as the consequence of chronic increased afterload.^32,33^ The more impairment of the LV, the more impact of PPM.^5^ In men, patients with left ventricular dysfunction will present with eccentric hypertrophy of the LV and reduced ejection fraction. As reduced ejection fraction is a major predictor of mortality post-surgical AVR, the vast majority of patients with reduced ejection fraction will be referred to transcatheter intervention. In women, ejection fraction increases with the concentric remodelling of the LV, and tends to shadow the impairment of the LV. In the present cohort, women presented smaller LV, higher relative wall thickness, and higher left ventricular ejection fraction, as expected given the sex-specific remodelling of the LV,^34^ which is not synonymous of healthier LV. Indeed, in patients with normal ejection fraction, women present, at any stage of aortic stenosis severity, more left ventricular fibrosis than men.^35^ Thus, despite being older at the time of AVR, a more impaired LV could explain a greater impact of PPM in women.

### Sex-specific management of aortic stenosis

Both men and women exhibit a major independent impact of PPM following the application of the refined definition. Consequently, any degree of PPM should be avoided in both sexes, considering sex-specific EOAi thresholds. Of note, PPM may have a greater impact amongst women than men. This is evidenced by the persistence of the independent association with cardiovascular mortality solely in women, even after optimizing the PPM definition.

As PPM is calculated by published EOA of the prosthesis, the possible occurrence of PPM could be predicted before intervention and thus a prothesis with better haemodynamic features or an enlargement of the aortic annulus could be planned. Finally, referral to transcatheter AVR could be preferred in women, which is currently evaluated in the Randomized researcH in womEn all comers wIth Aortic stenosis (RHEIA) trial.^36^

Regarding the follow-up of patients with PPM, PPM should probably be addressed as native aortic stenosis, meaning that a severe PPM in a symptomatic patient or in a patient with an impaired LV should be treated if better results could be achieved. Moreover, the haemodynamic obstruction associated with moderate PPM will progress with the association with thrombosis, pannus, or bioprosthesis degeneration. This progression is being accelerated by the PPM itself.^37,38^ Thus, the absence of echocardiographic follow-up during the first 5 years post-surgery^39^ in these patients should probably be revised.

### Limitations

This study was an observational study encompassing a limited geographic area (region of Québec, Canada), conducted in a centre with concomitant high-volume transcatheter AVR, thus the low incidence of PPM after AVR should not be generalized to the general population. However, this study encompassed consecutive patients undergoing AVR in the time frame from 2001 to 2021, the broader in the literature to the best of our knowledge. Furthermore, the low incidence of severe PPM does not allow for a specific analysis in this subgroup of patients without losing statistical power (1.1% of patients). Multicenter registries are warranted to explore machine learning-based algorithms for predicting outcome after surgical AVR considering the complete baseline individual patient profile.

## Conclusions

In this large series of AVR patients, the incidence of severe PPM was low (<2%). Prosthesis-patient mismatch was more prevalent in women vs. men, and was independently associated with increased risk of long-term mortality only in women, according to the VARC-3 definition. Refining PPM with sex-specific thresholds identified it as an independent predictor of mortality in men within our cohort, suggesting that higher EOAi cut-offs may be required for men compared with women.

While our findings underscore important clinical implications for managing aortic valve disease, particularly in addressing sex differences, these conclusions must be interpreted within the context of a single, highly specialized centre with a high volume of transcatheter AVR procedures, which may explain the low incidence of severe PPM. Nevertheless, our study provides valuable insights into PPM and sex differences, currently underexplored in the existing literature.

These results emphasize the need for further research to refine aortic valve intervention strategies, especially for women, to mitigate the adverse effects of PPM.

## Supplementary Material

ehaf076_Supplementary_Data

## Declarations

### Data Availability

The data that support the findings of this study are available on request from the corresponding author [M.-A.C.], after evaluation and agreement by our local ethics committee.

### Pre-registered Clinical Trial Number

None supplied.
