# Supplementary material for: Sex-related differences in prosthesis-patient mismatch after surgical aortic valve replacement and long-term outcomes
Source: Eur Heart J. 2025 Feb 18;46(24):2320–32. doi: 10.1093/eurheartj/ehaf076 (PMC12187514; doi:10.1093/eurheartj/ehaf076)
Supplement: ehaf076_Supplementary_Data [file ehaf076_supplementary_data.pdf]

Supplemental material for

Sex difference in Prosthesis-Patient Mismatch after Surgical Aortic Valve Replacement and long-term clinical outcome

Paolo Springhetti, MD; Kathia Abdoun, MSc; Éric Dumont, MD; François Dagenais, MD; Dimitri Kalavrouziotis, MD; Siamak Mohammadi, MD; Philippe Pibarot, DVM, PhD; Marie-Annick Clavel, DVM, PhD

Supplementary content:

Table S1. Predicted EOAs for each prosthesis model with references. (Page 3)

Table S2. Multivariable Cox-Regression of Cardiovascular Mortality in Women (PPM defined according to VARC 3 Definition and Spline Curve Thresholds*). (Page 4)

Table S3 Multivariable Cox-Regression of Cardiovascular Mortality in Men (PPM defined by VARC 3 Definition vs. Spline Curve Thresholds). (Page 5)

Table S4. Logistic regression analysis for perioperative mortality in women. (Page 6)

Table S5. Logistic regression analysis for perioperative mortality in men. (Page 6)

Table S6. Baseline clinical features of the study cohort disaggregated by sex and age (elderly - ≥ 70 years vs non-elderly - < 70 years). (Pages 7 - 8)

Figure S1. Correlation matrix in different clinical baseline features and presence of Patients-Prosthesis Mismatch, according to VARC3 (Panel A) and to spline-derived refined definition (Panel B). (Pages 9 – 10)

Figure S2. Incidence of Prosthesis-Patients mismatch according to the year of intervention. (Page 11)

Figure S3. Histograms representing the Prosthesis-Patients mismatch incidence according to the year of intervention, disaggregating by sex. (Page 12)

Figure S4. Linear regression model representing the predicted PPM incidence over time, disaggregating by sex. (Page 13)

The slope of the two curves are not statistically different (p=0.83).

Figure S5. Calibration Plot adopting Hosmer-Lemeshow test. Predicted probabilities were grouped into deciles (tentiles), with approximately equal numbers of observations in each group. The test compares observed and expected events within each decile, and a non-significant p-value indicates good model calibration for all-cause mortality (pannel A at 10 years, pannel B at 15 years) and CV mortality (pannel C at 10 years, pannel D at 15 years). (Page 14)

Figure S6. Time ROC curve for all-cause mortality at 15 years, in women (Panel A) and men (Panel B). (Pages 15)

In men, Model 1 includes the VARC3 criteria and Model 2 the spline-derived refined definition of PPM.

Figure S7. Kaplan Meyer analysis stratified for PPM – refined definition - and Age, in men (Panel A) and women (Panel B). (Pages 16 – 17)

Figure S8. The normogram displaying the predicted 10-year survival after SAVR from the Cox multivariable analysis of death for different Age in relation to EOAi as a continuous variable. (Page 18)

Table S1: Predicted EOAs for each prosthesis model with references

	Size						Reference
**Model**	**19 (18*) (S**)**	**21 (20*) (M**)**	**23 (24*) (L**)**	**25 (XL**)**	**27 (26*)**	**29**	
St Jude mechanical master series	1	1.4	1.5	2.1	2.7	3.2	[1]
St Jude Regent	1	1.3	1.6	1.9	2.3	2	[2]
Mcri On X	1.5	1.7	2.0	2.4	3.2	3.2	[1]
MedAdvantage	1.2	1.7	2.1	2.4	2.9	3.2	[3]
Carbomedics Top Hat	1	1.5	1.7	2.0	2.5	2.6	[1]
ATS AP *	1.1	1.4	1.9	2.1	2.3	NA	[4]
Mitroflow	1.1	1.30	1.50	1.80	NA	NA	[5]
Perimount 2900	1.1	1.30	1.50	1.80	2.10	NA	[2]
Freestyle	1.2	1.4	1.5	2.0	2.3	2.6	[1] [6]
Mosaic	1.1	1.22	1.38	1.65	1.59	2.0	[7,8]
St Jude Toronto		1.2	1.5	1.9	2.3	2.6	[9]
Cryolife Obrien	NA	1.2	1.6	2	2	NA	[10]
Perimount Magna	1.3	1.70	2.10	2.30	NA		[8,11]
Epic	1	1.1	1.3	1.5	1.7	2.4	[12]
Mosaic Ultra	1.1*	1.2	1.6	1.8			[13]
Perimount Magna Ease	1.2	1.50	1.80	1.80	2.10		[14,15]
Trifecta	1.4	1.60	1.80	2.00	2.20		[16]
Solo Freedom	1.0	1.3	1.6	1.8	2.0		[17]
Enable Med	1.9	2.1	2.0	2.1	2.4		[18]
Perceval**	1.5	1.70	1.80	2.10	NA		[19]
Perigon Med	1.1	1.3	1.5	1.6	1.8	2.0	[20]
Intuity	1.1	1.30	1.70	1.90	2.20		[21]
Resilia Inspiris	1.1	1.3	1.5	1.7	2.3		[22]

* ATS AP sizes: 18mm, 20mm, 24mm, 25mm, 26mm

** Perceval sizes: S, M, L, XL.

NA = Not available

<u>Table S2.</u> Multivariable Cox-Regression of Cardiovascular Mortality in Women (PPM defined according to VARC 3 Definition and Spline Curve Thresholds*)

	HR (95% CI)	p-value
Age (1 year increase)	1.06 (1.05 – 1.08)	**<0.0001**
BMI (1 unit increase)	1.01 (0.99 – 1.03)	0.09
Atrial Fibrillation	1.92 (1.54 – 2.40)	**<0.0001**
Previous Stroke or TIA	1.17 (0.89 – 1.55)	0.26
Diabetes	1.74 (1.43 – 2.10)	**<0.0001**
Hypertension	1.12 (0.89 – 1.41)	0.32
Dyslipidemia	1.18 (0.95 – 1.47)	0.13
COPD	1.46 (1.14 – 1.89)	**0.003**
CAD	1.53 (1.26 – 1.85)	**<0.0001**
CKD	1.41 (0.80 – 2.46)	0.22
Smoking status	1.37 (0.99 – 1.90)	0.06
NYHA III/IV	1.06 (0.89 – 1.27)	0.49
Angina CCS 4/5	1.18 (0.89 – 1.56)	0.23
LVEF (1% unit increase)	0.50 (0.26 – 0.95)	**0.04**
Mechanical prosthesis	1.27 (0.90 – 1.77)	0.17
PPM	1.21 (1.01 - 1.46)	**0.04**

BMI body mass index, CCS, Canadian Cardiovascular Society angina grading, NYHA New York Heart Association, COPD chronic obstructive pulmonary disease, CAD coronary artery disease, CKD Chronic Kidney Disease, LVEF left ventricle ejection fraction, TIA, transient ischemic attack, PPM, Prosthesis-patient mismatch. *VARC 3 definition-Effective Orifice Area Index thresholds align to those derived by Spline Curve Analysis, so the model does not change.

<u>Table S3.</u> Multivariable Cox-Regression of Cardiovascular Mortality in Men (PPM defined by VARC 3 Definition vs. Spline Curve Thresholds)

	**Model 1**<br>**VARC 3 Definition**		**Model 2**<br>**Spline Curve Thresolds***	
	HR (95% CI)	p-value	HR (95% CI)	p-value
Age (1 year increase)	1.06 (1.05 – 1.08)	**<0.0001**	1.06 (1.05 – 1.08)	**<0.001**
BMI (1 unit increase)	1.01 (1.00 – 1.03)	0.12	1.01 (0.99 – 1.03)	0.19
Atrial Fibrillation	1.92 (1.53 – 2.40)	**0.0004**	1.42 (1.17 – 1.72)	**0.0003**
Previous Stroke or TIA	1.56 (1.26 – 1.94)	**<0.0001**	1.56 (1.23 – 1.93)	**<0.0001**
Diabetes	1.39 (1.18 – 1.64)	**<0.0001**	1.38 (1.16 – 1.65)	**<0.0001**
Hypertension	1.01 (0.85 – 1.20)	0.89	1.01 (0.85 – 1.20)	0.89
Dyslipidemia	1.28 (1.05 – 1.56)	**0.01**	1.28 (1.05 – 1.55)	**0.01**
COPD	1.57 (1.28 – 1.91)	**<0.0001**	1.57 (1.28 – 1.91)	**<0.0001**
CAD	1.62 (1.37 – 1.93)	**<0.0001**	1.62 (1.37 – 1.92)	**<0.0001**
CKD	2.65 (2.07 – 3.41)	**<0.0001**	2.67 (2.06 – 3.41)	**<0.0001**
Smoking status	1.26 (0.99 – 1.59)	0.06	1.26 (0.99- 1.59)	0.06
NYHA III/IV	1.11 (0.96 – 1.31)	**<0.16**	1.12 (0.96 – 1.31)	0.15
Angina CCS 4/5	1.09 (0.89 – 1.33)	0.43	1.08 (0.88 – 1.32)	0.45
LVEF (1% increase)	0.98 (0.97 – 0.98)	**<0.0001**	0.98 (0.97 – 0.98)	**<0.0001**
Mechanical prosthesis	1.15 (0.87 – 1.52)	0.33	1.15 (0.87 – 1.53)	0.31
PPM	1.05 (0.87 - 1.27)	0.66	1.14 (0.98-1.33)	0.09

BMI body mass index, CCS, Canadian Cardiovascular Society angina grading, NYHA New York Heart Association, COPD chronic obstructive pulmonary disease, CAD coronary artery disease, CKD Chronic Kidney Disease, LVEF left ventricle ejection fraction, TIA, transient ischemic attack, PPM, Prosthesis-patient mismatch. *Effective Orifice Area Indexed (EOAi)<0.90 if BMI <30 kg/m2, EOAi<0.80 if BMI ≥ 30 kg/m2.

Table S4. Logistic regression analysis for perioperative mortality in women.

	VARC 3 Definition*	
	OR (95% CI)	p-value
EuroScore II	1.09 (1.06 - 1.11)	**<0.0001**
PPM	1.52 (1.01 – 2.30)	**0.04**

Abbreviations:

PPM, Prosthesis-patient mismatch. *VARC 3 definition-Effective Orifice Area Index thresholds align to those derived by Spline Curve Analysis, so the model does not change.

Table S5. Logistic regression analysis for perioperative mortality in men.

	VARC 3 Definition		Spline Curve Thresolds	
	OR (95% CI)	p-value	OR (95% CI)	p-value
EuroScore II	1.10 (1.08 – 1.11)	**<0.0001**	1.09 (1.08 – 1.11)	**<0.0001**
PPM	1.19 (0.72 – 1.95)	0.50	1.67 (1.11 – 2.53)	**0.01**

Abbreviations:

PPM, Prosthesis-patient mismatch.

Table S6. Baseline clinical features of the study cohort disaggregated by sex and age (elderly - ≥ 70 years vs non-elderly - < 70 years).

	**Male n=4,993**			**Female n=2,326**		
	<70 years (n=2545; 51.0%)	≥ 70 years (n=2448; 49.0%)	P value	<70 years (n=838; 36.0%)	≥ 70 years (n=1488; 63.9%)	P value
Age (years)	60.3 ± 7.9	76.3 ± 4.5	**<0.0001**	61.1 ± 7.3	77.4 ± 4.6	**<0.0001**
BSA (mq)	2.00 ± 0.21	1.92 ± 0.19	**<0.0001**	1.77 ± 0.21	1.69 ± 0.19	**<0.0001**
BMI (kg/mq)	28.8 ± 5.4	27.6 ± 4.4	**<0.0001**	29.3 ± 6.5	27.9 ± 5.6	**<0.0001**
Diabetes	617 (24.2%)	751 (30.7%)	**<0.0001**	212 (25.2%)	409 (27.4%)	0.25
Hypertension	1655 (65.0%)	1902 (77.7%)	**<0.0001**	549 (65.5%)	1187 (79.8%)	**<0.0001**
Dyslipidemia	1995 (78.4%)	2079 (84.9%)	**<0.0001**	660 (78.7%)	1174 (78.9%)	0.93
Atrial fibrillation	232 (9.1%)	456 (18.6%)	**<0.0001**	62 (7.4%)	267 (17.9%)	**<0.0001**
Coronary artery disease	1117 (43.8%)	1659 (67.8%)	**<0.0001**	224 (26.7%)	643 (43.2%)	**<0.0001**
Previous myocardial infarction	433 (17.0%)	570 (23.3%)	**<0.0001**	81 (9.7%)	216 (14.5%)	**0.0007**
COPD	225 (8.8%)	346 (14.1%)	**<0.0001**	80 (9.5%)	185 (12.4%)	**0.04**
Smoking status	449 (17.6%)	153 (6.2%)	**<0.0001**	150 (17.1%)	85 (5.7%)	**<0.0003**
Previous stroke or TIA	193 (7.5%)	271 (11.1%)	**<0.001**	65 (7.7%)	145 (9.7%)	0.14
CKD	63 (2.4%)	134 (5.4%)	**<0.0001**	12 (1.4%)	32 (2.1%)	0.27
eGFR Cockroft (ml/min/m^2)	85.9 ± 24.0	61.1 ± 17.9	**<0.0001**	72.7 ± 21.9	50.3 ± 15.7	**<0.0001**
NYHA III or IV	825 (32.4%)	922 (37.7%)	**0.0001**	382 (45.5%)	730 (49.0%)	0.11
Angina (CCS 4 or 5)	391 (15.4%)	540 (22.2%)	**<0.0001**	93 (11.1%)	234 (15.7%)	**0.002**
Euroscore II	1.97 (1.07-3.55)	3.30 (1.99-6.01)	**<0.0001**	2.11 (1.26 – 3.96)	3.95 (2.46 – 6.83)	**<0.0001**
**Echocardiographic data**						

LVEF (%)	56.7 ± 12.0	56.7 ± 11.1	0.52	60.4 ± 10.1	60.2 ± 10.6	0.42
Left atrium index (ml/m^2)	39.9 ± 7.6	42.3 ± 7.0	**<0.0001**	36.9 ± 6.0	39.7 ± 6.7	**<0.0001**
LVEDd (mm)	51.1 ± 7.9	48.6 ± 6.9	**<0.0001**	45.6 ± 6.5	43.9 ± 6.4	**<0.0001**
LVMi (g/m^2)	117.6 ± 39.4	112.2 ± 36.8	**<0.0001**	98.3 ± 32.0	99.7 ± 32.0	0.78
RWT	0.46 ± 0.12	0.48 ± 0.12	**<0.0001**	0.47 (0.39-0.53)	0.49 (0.42-0.67)	**0.0004**
Peak Gradient (mmHg)	65.6 ± 31.2	64.7 ± 26.8	0.18	42.9 ± 18.9	42.6 ± 18.4	0.78
Mean Gradient (mmHg)	39.8 ± 19.8	38.9 ± 16.6	0.07	70.5 ± 28.9	70.1 ± 28.6	0.77
**Operative data**						
Valve Size (mm)	24.6 ± 2.0	24.1 ± 1.8	**<0.0001**	21.8 ± 1.8	21.5 ± 1.7	**0.0001**
Mechanical prosthesis	626 (24.5%)	22 (0.9%)	**<0.0001**	215 (25.6%)	28 (1.9%)	**<0.0001**

Data are expressed as mean ± standard deviation or median (interquartile-range). *Obtained with T-Test or Wilcoxon-Mann-Whitney test for continuous variables as appropriate, or Chi Square for categorical variables.

BMI body mass index, BSA, body surface area (Mosteller method), CCS, Canadian Cardiovascular Society angina grading, NYHA New York Heart Association, COPD chronic obstructive pulmonary disease, CAD coronary artery disease, CKD Chronic Kidney Disease, eGFR, estimated Glomerular Filtration Rate (Cockroft formula), LVEDd, Left ventricular end diastolic diameter, LVEF left ventricle ejection fraction, LVMI left ventricular mass index, RWT relative wall thickness, TIA, transient ischemic atta

<u>Figure S1</u> – Panel A. Correlation matrix in different clinical baseline features and presence of Patients-Prosthesis Mismatch (according to VARC3– Panel A, and refined spline-derived definition – Panel B).

A.

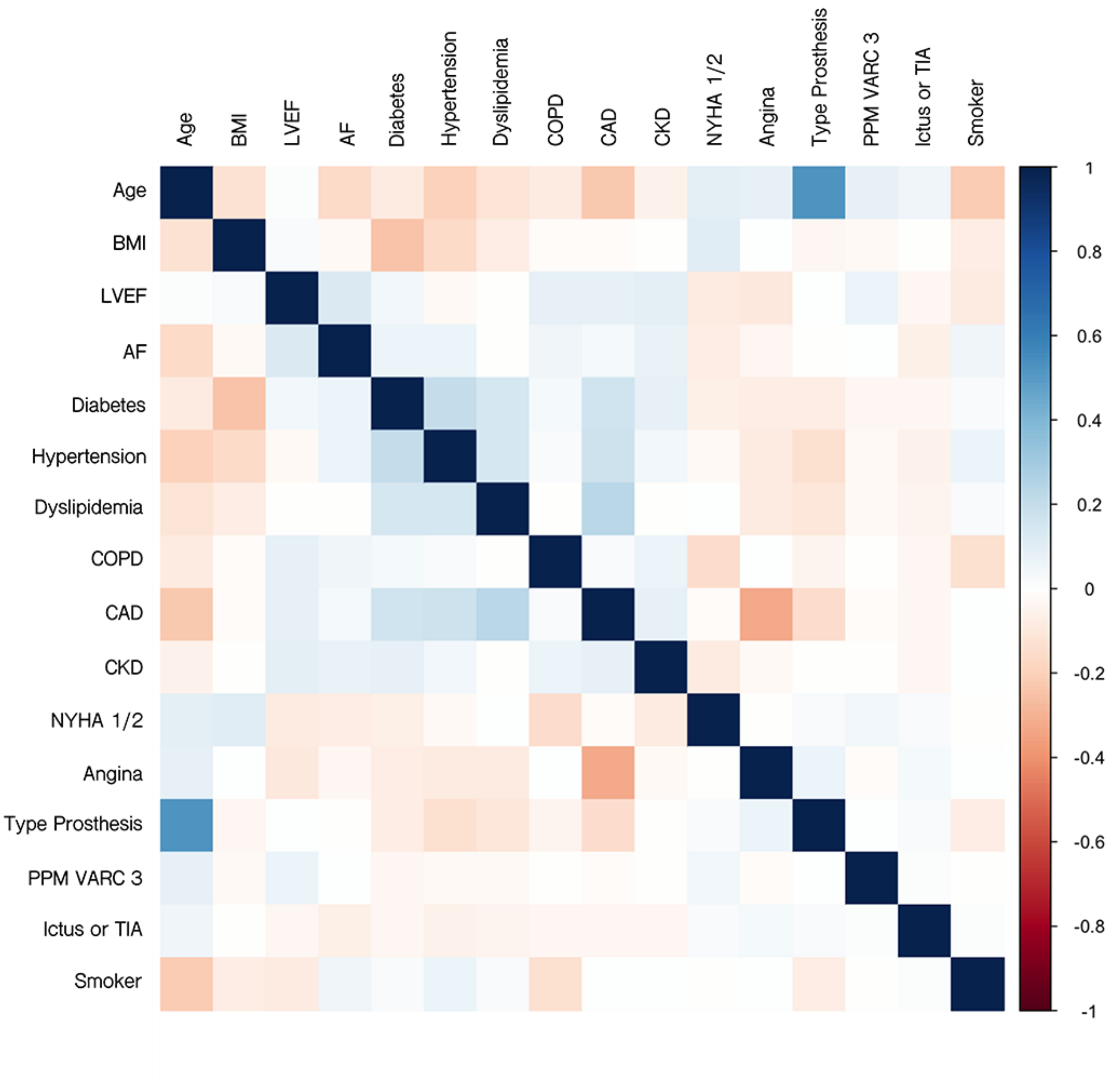

B.

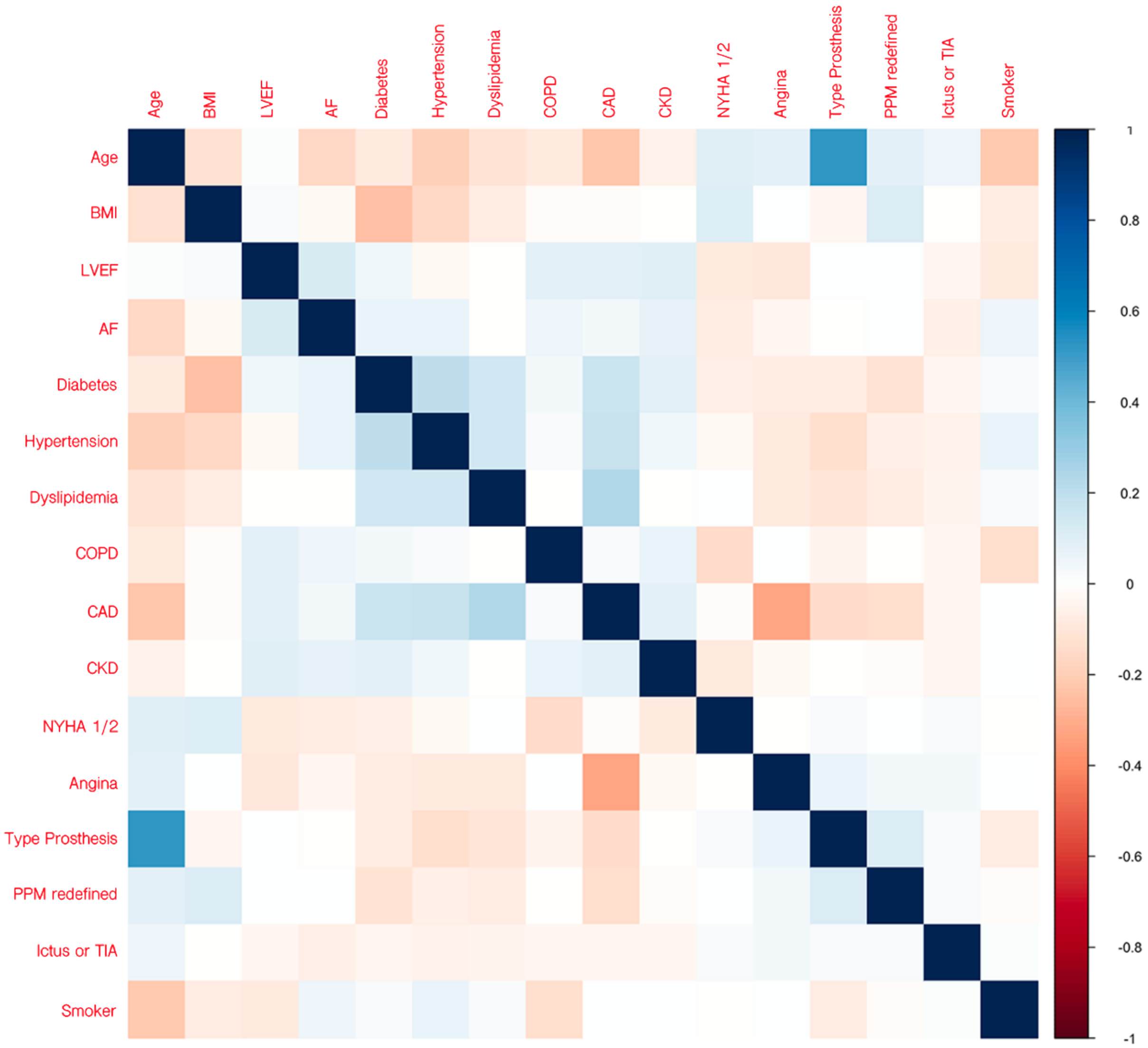
Age
BMI
LVEF
AF
Diabetes
Hypertension
Dyslipidemia
COPD
CAD
CKD
NYHA 1/2
Angina
Type Prosthesis
PPM redefined
Ictus or TIA
Smoker
1
0.8
0.6
0.4
0.2
0
-0.2
-0.4
-0.6
-0.8
-1

<u>Figure S2.</u> Incidence of Prosthesis-Patients mismatch according to the year of intervention

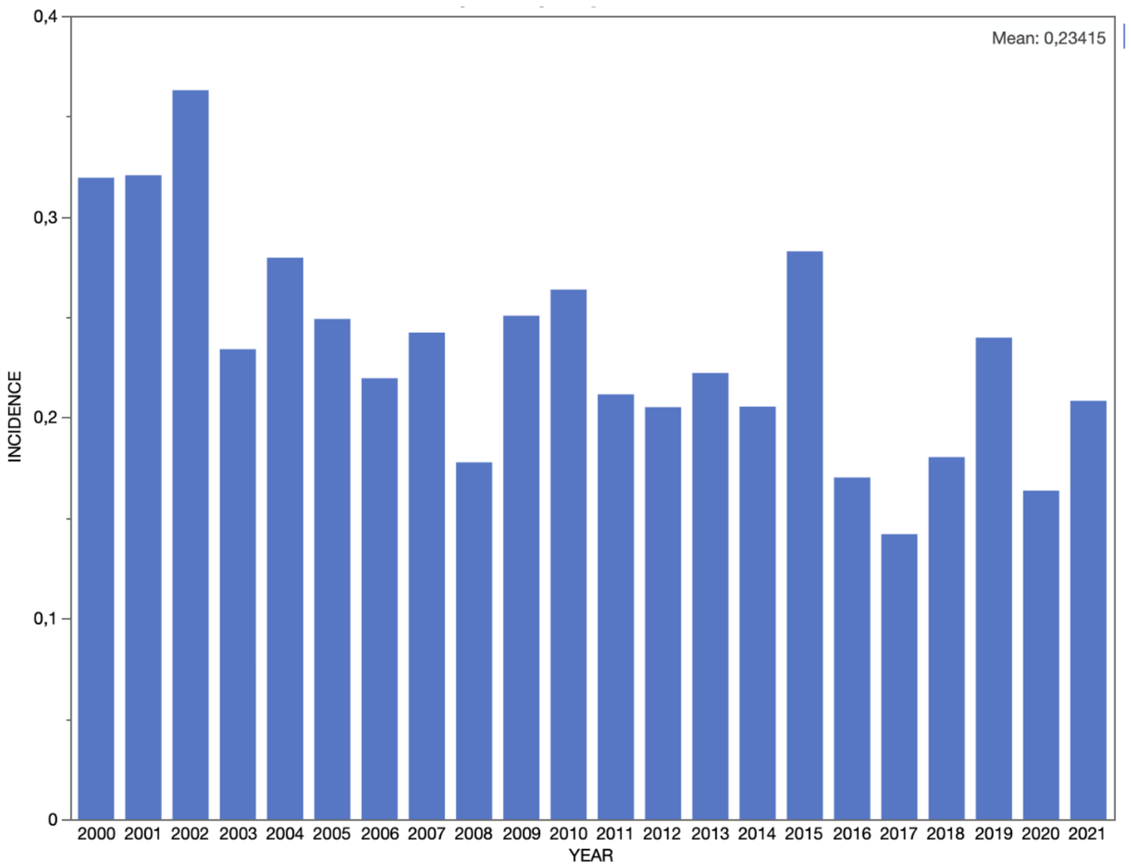

<u>Figure S3</u> Incidence of Prosthesis-Patients mismatch according to the year of intervention, in men (Panel A), and women (Panel B)

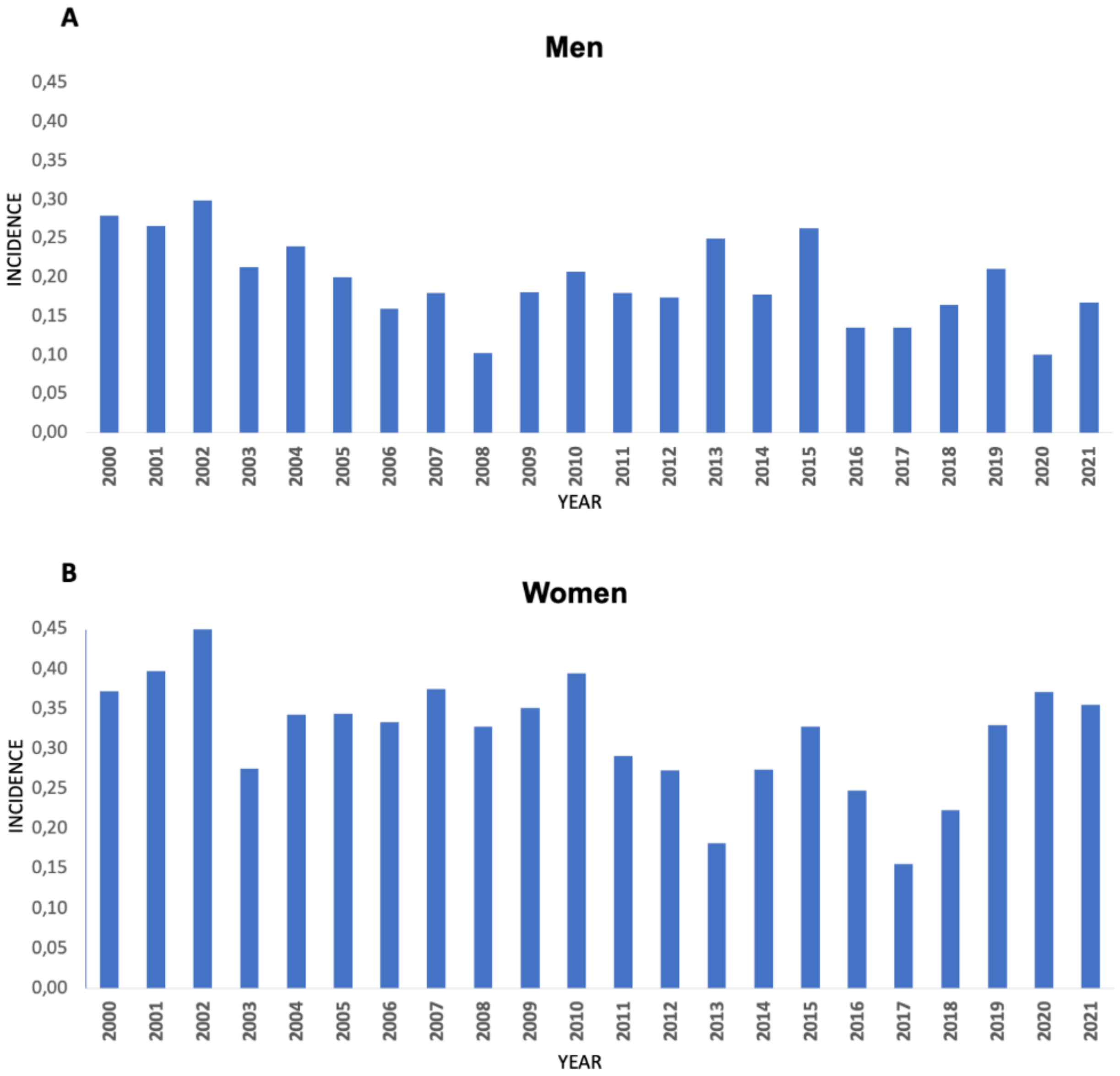

Figure S4. Linear regression model representing the predicted PPM incidence over time, disaggregating by sex. The slope of the two curves is not statistically different (p=0.83).

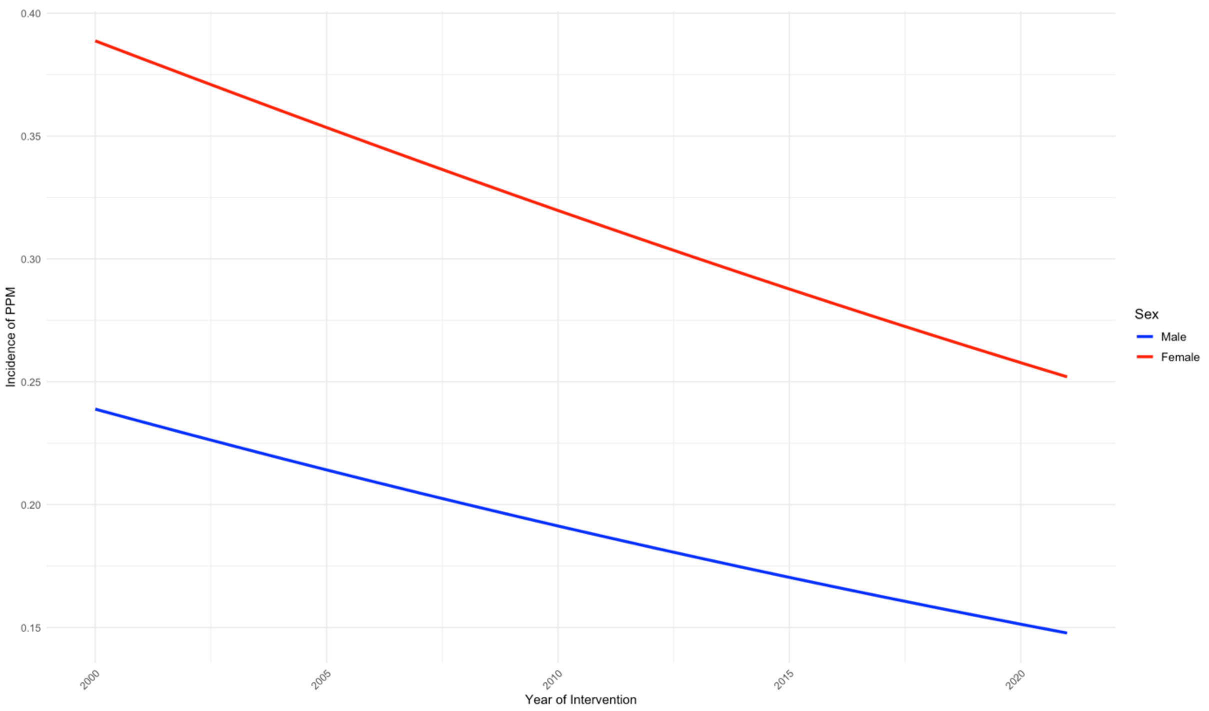

<u>Figure S5.</u> Calibration Plot adopting Hosmer-Lemeshow test. Predicted probabilities were grouped into deciles (tentiles), with approximately equal numbers of observations in each group. The test compares observed and expected events within each decile, and a non-significant p-value indicates good model calibration for all-cause mortality (pannel A at 10 years, pannel B at 15 years) and CV mortality (pannel C at 10 years, pannel D at 15 years).

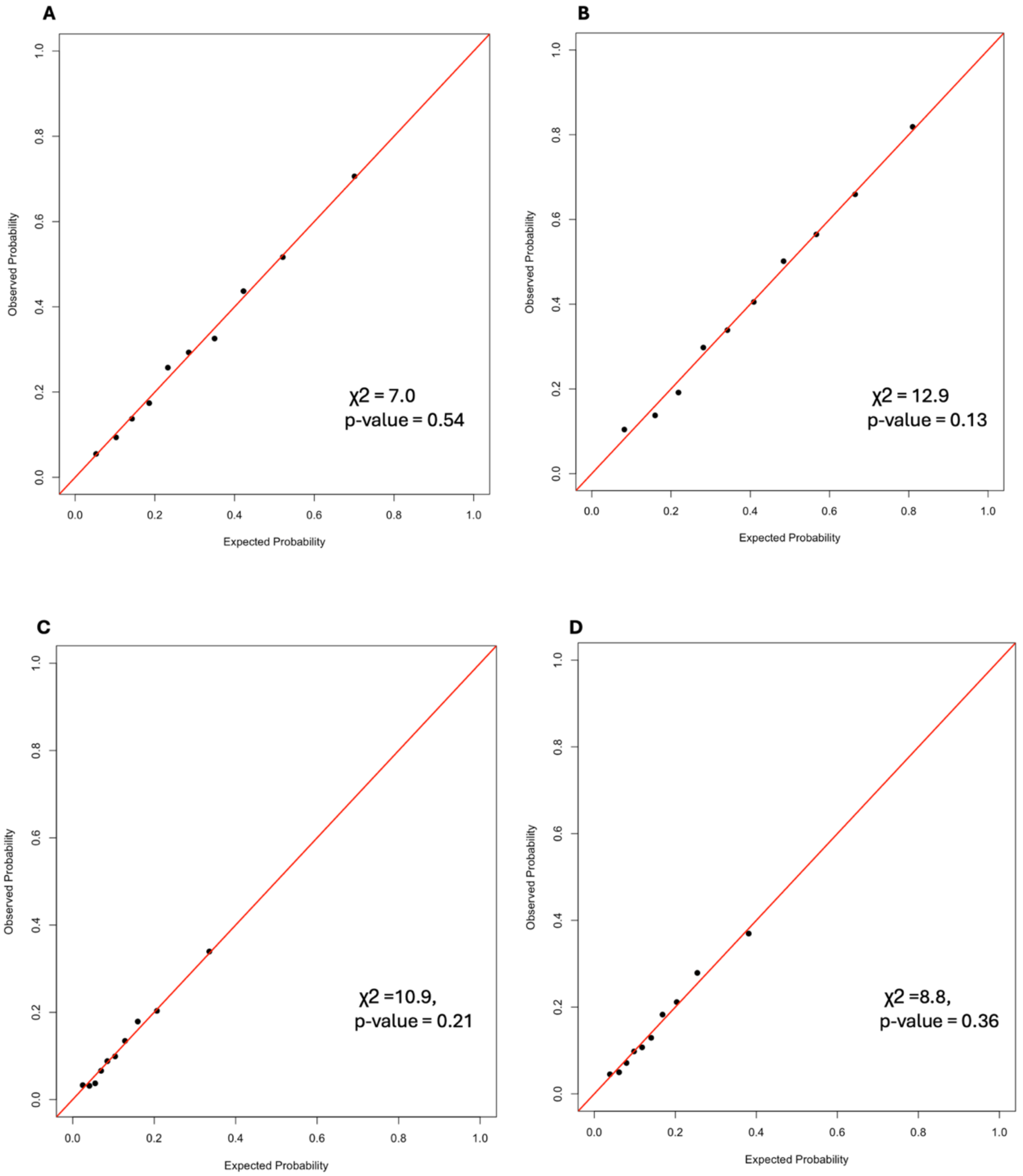

<u>Figure S6 -</u> Time ROC curve for all-cause mortality at 15 years, in women (Panel A) and men (Panel B). In men, Model 1 includes the VARC3 criteria and Model 2 the spline-derived refined definition of PPM.

A

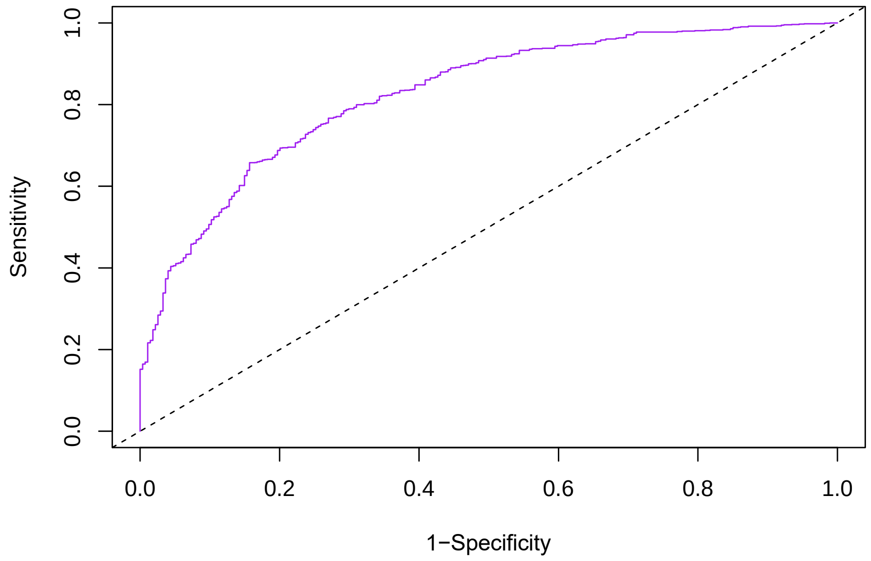

B

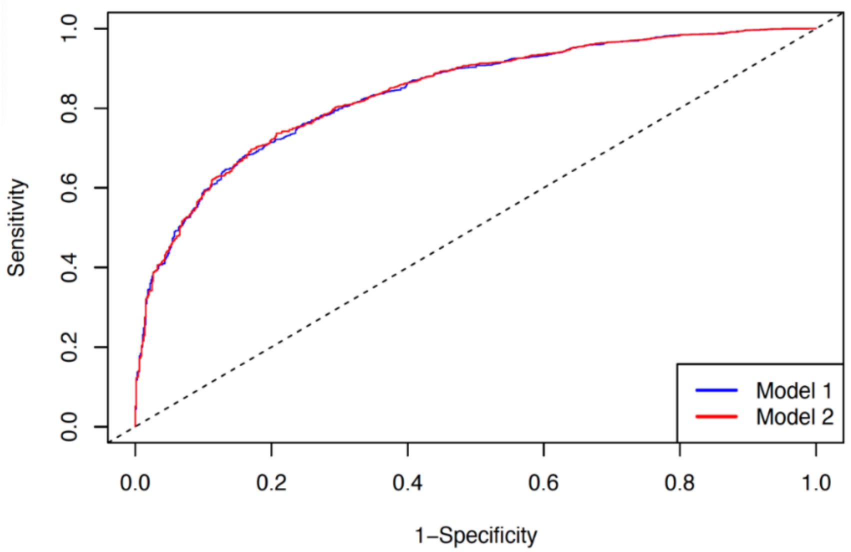

<u>Figure S7.</u> Kaplan Meyer analysis stratified for PPM – refined definition - and Age, in men (Panel A) and women (Panel B).

<u>Panels A</u>

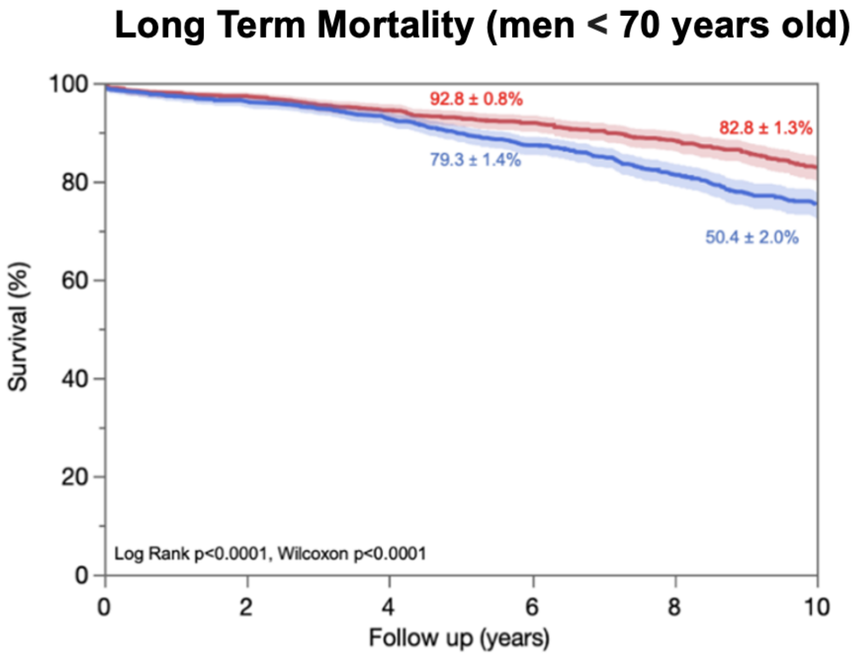

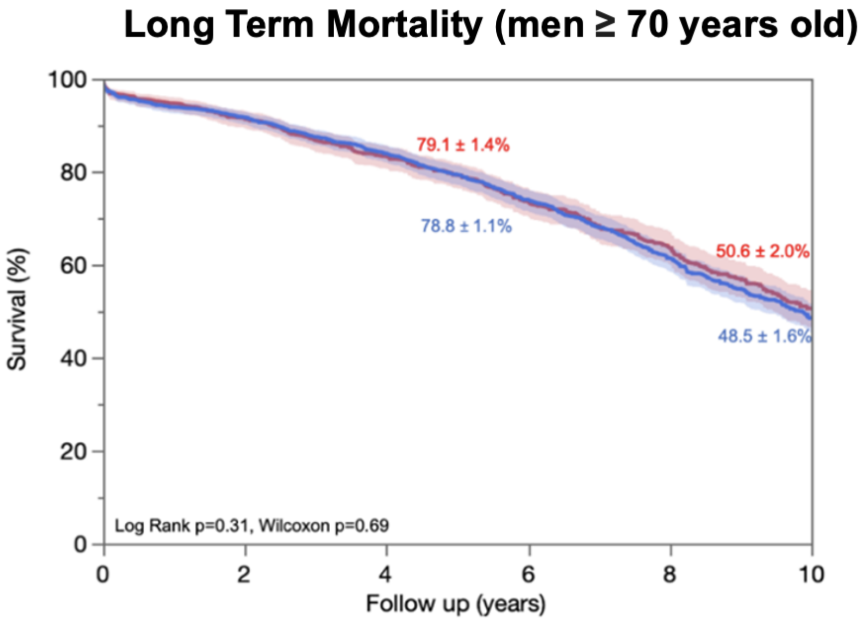

Panels B

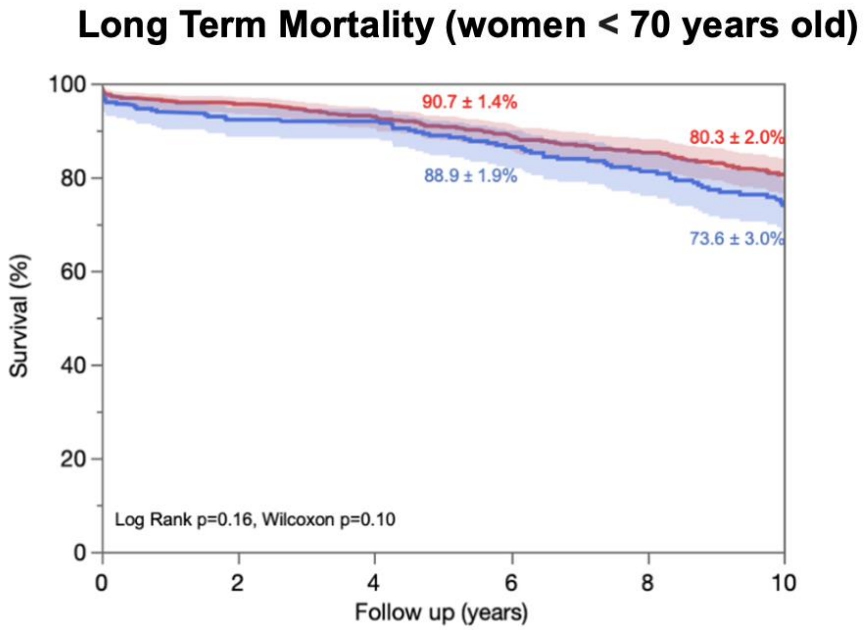
Long Term Mortality (women < 70 years old)
90.7 ± 1.4%
80.3 ± 2.0%
88.9 ± 1.9%
73.6 ± 3.0%
Survival (%)
Follow up (years)
Log Rank p=0.16, Wilcoxon p=0.10
100
80
60
40
20
0
0
2
4
6
8
10

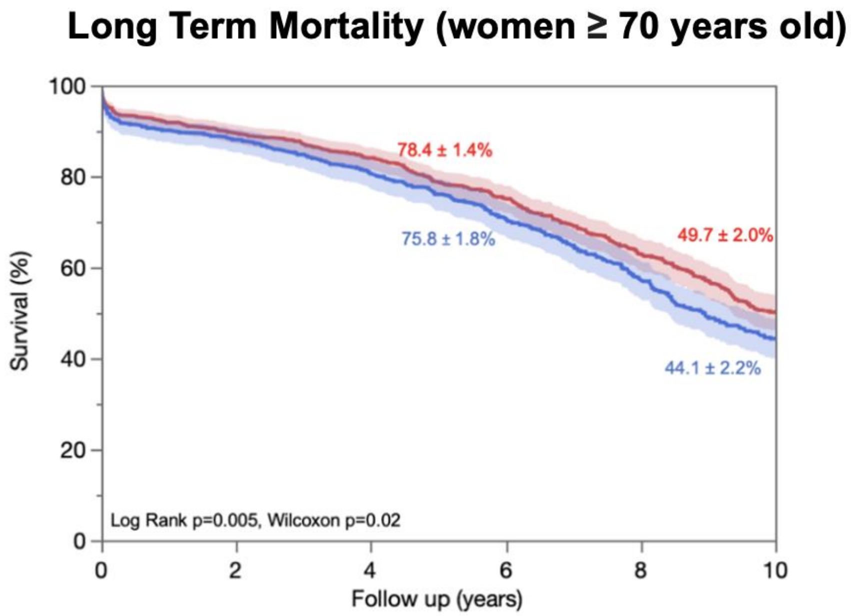
Long Term Mortality (women ≥ 70 years old)
78.4 ± 1.4%
75.8 ± 1.8%
49.7 ± 2.0%
44.1 ± 2.2%
Survival (%)
Follow up (years)
Log Rank p=0.005, Wilcoxon p=0.02
100
80
60
40
20
0
0
2
4
6
8
10

<u>Figure S8.</u> The normogram displaying the predicted 10-year survival after SAVR from the Cox multivariable analysis of death for different Age in relation to EOAi as a continuous variable (cm^2/m^2).

To obtain the risk-adjusted curve, the values for the following variables were maintained fixed: NYHA class 1 or 2, BMI=25, no previous history of TIA/ictus/hypertension/kidney disease/COPD/dysplipidemia/CAD/angina, bioprosthetic aortic valve and year of intervention=2018. Confidence intervals at 95% are displayed.

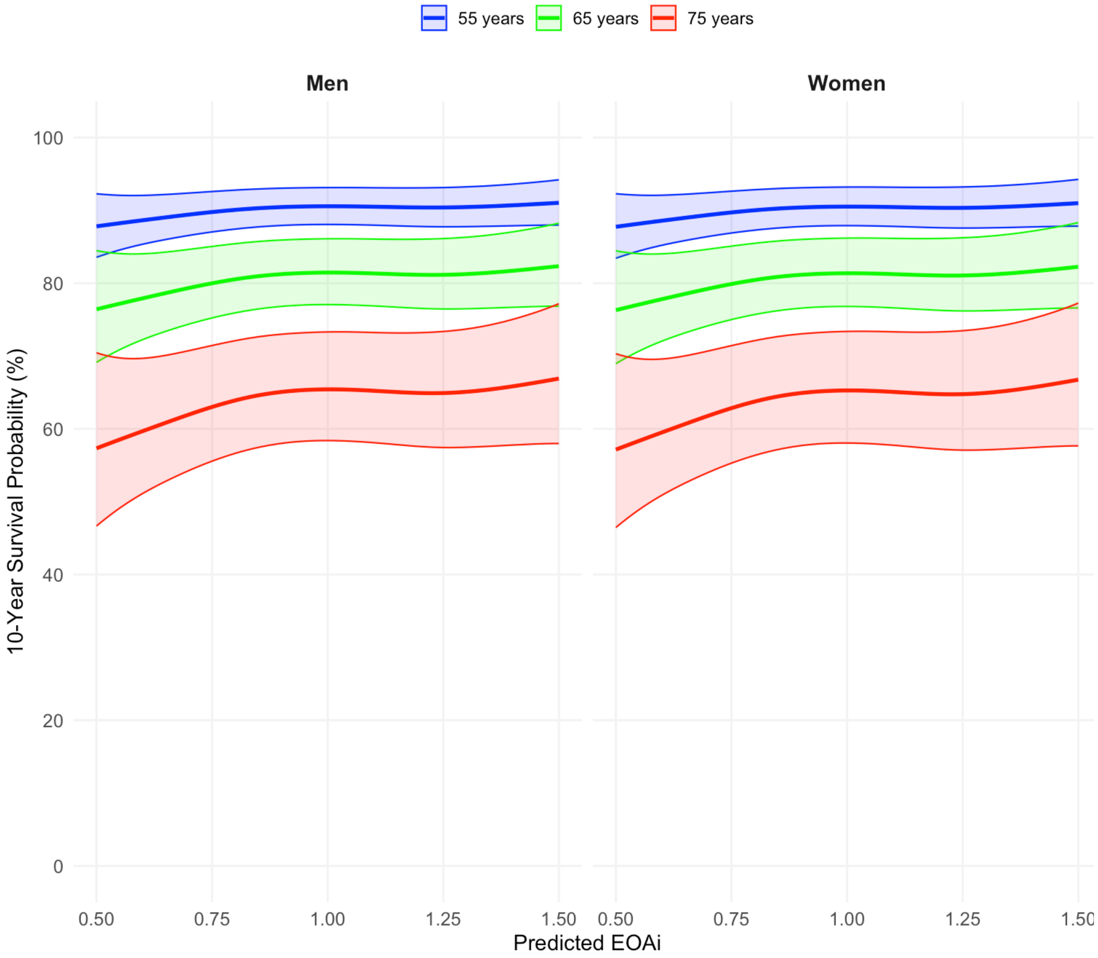

References:

1. Lancellotti P, Pibarot P, Chambers J et al Recommendations for the imaging assessment of prosthetic heart valves: a report from the European Association of Cardiovascular Imaging endorsed by the Chinese Society of Echocardiography, the Inter-American Society of Echocardiography, and the Brazilian Department of Cardiovascular Imaging. Eur Heart J Cardiovasc Imaging. 2016 Jun;17(6):589-90.
2. Bach DS, Sakwa MP, Goldbach M, Petracek MR, Emery RW, Mohr FW. Hemodynamics and early clinical performance of the St. Jude Medical Regent mechanical aortic valve. Ann Thorac Surg. 2002 Dec;74(6):2003-9.
3. Haaverstad R, Vitale N, Karevold A, Cappabianca G, Tromsdal A, Olsen PS et al. Clinical and echocardiographic assessment of the Medtronic Advantage aortic valve prosthesis: the Scandinavian multicentre, prospective study. Heart. 2007 Apr;93(4):500-5.
4. Emery RW, Van Nooten GJ, Tesar PJ; Investigators for the ATS Clinical Open Pivot Heart Valve Food and Drug Administration Study. The initial experience with the ATS Medical mechanical cardiac valve prosthesis. Ann Thorac Surg. 2003 Feb;75(2):444-52.
5. García-Bengochea J, Sierra J, González-Juanatey JR, Rubio J, Vega M, Fernández AL, et al. Left ventricular mass regression after aortic valve replacement with the new Mitroflow 12A pericardial bioprosthesis. J Heart Valve Dis 2006;15:446–51.
6. Yun KL, Sintek CF, Fletcher AD, Pfeffer TA, Kochamba GS, Hyde MR et al. Aortic valve replacement with the freestyle stentless bioprosthesis: five-year experience. Circulation. 1999 Nov 9;100(19 Suppl):II17-23.
7. Pibarot P, Dumesnil JG. Hemodynamic and clinical impact of prosthesis–patient mismatch in the aortic valve position and its prevention. J Am Coll Cardiol 2000;36:1131–41.
8. Dalmau MJ, Maria Gonzalez-Santos J, Lopez-Rodriguez J, Bueno M, Arribas A, Nieto F. One year hemodynamic performance of the Perimount Magna pericardial xenograft and the Medtronic Mosaic bioprosthesis in the aortic position: a prospective randomized study. Interact Cardiovasc Thorac Surg 2007;6:345–9.
9. Gleason TG, David TE, Coselli JS, Hammon JW Jr, Bavaria JE. St. Jude Medical Toronto biologic aortic root prosthesis: early FDA phase II IDE study results. Ann Thorac Surg. 2004 Sep;78(3):786-93.
10. Chambers JB, Rimington HM, Rajani R, Hodson F, Shabbo F. A randomized comparison of the Cryolife O'Brien and Toronto stentless replacement aortic valves. J Thorac Cardiovasc Surg. 2007 Apr;133(4):1045-50.
11. Botzenhardt F, Eichinger WB, Guenzinger R, Bleiziffer S, Wagner I, Bauernschmitt R, et al. Hemodynamic Performance and Incidence of Patient-Prosthesis Mismatch of the Complete Supraannular Perimount Magna Bioprosthesis in the Aortic Position. Thorac Cardiovasc Surg 2005;53:226–30.
12. Dellgren G, Eriksson MJ, Brodin LA, Rådegran K. Eleven years' experience with the Biocor stentless aortic bioprosthesis: clinical and hemodynamic follow-up with long-term relative survival rate. Eur J Cardiothorac Surg. 2002 Dec;22(6):912-21.

13. Ruzicka DJ, Hettich I, Hutter A, Bleiziffer S, Badiu CC, Bauernschmitt R, Lange R, Eichinger WB. The complete supraannular concept: in vivo hemodynamics of bovine and porcine aortic bioprostheses. Circulation. 2009 Sep 15;120(11 Suppl):S139-45.

14. Mayr B, Burri M, Vitanova K, Prinzing A, Goppel G, Krane M, Lange R, Günzinger R. Serial echocardiographic evaluation of the Perimount Magna Ease prosthesis. J Thorac Dis. 2021 Jul;13(7):4104-4113.

15. Bavaria JE, Griffith B, Heimansohn DA, Rozanski J, Johnston DR, Bartus K, Girardi LN, Beaver T, Takayama H, Mumtaz MA, Rosengart TK, Starnes V, Timek TA, Boateng P, Ryan W, Cornwell LD, Blackstone EH, Borger MA, Pibarot P, Thourani VH, Svensson LG, Puskas JD; COMMENCE Trial Investigators. Five-year Outcomes of the COMMENCE Trial Investigating Aortic Valve Replacement With RESILIA Tissue. Ann Thorac Surg. 2023 Jun;115(6):1429-1436.

16. Deutsch M-A, Prinzing A, Fiegl K, Wottke M, Badiu CC, Krane M, et al. Early haemodynamic performance of a latest generation supra-annular aortic bioprosthesis: experience from a large single-centre series. Eur J Cardiothorac Surg. 2016 Jun;49(6):1691-8.

17. Heimansohn D, Roselli EE, Thourani VH, Wang S, Voisine P, Ye J et al. North American trial results at 1 year with the Sorin Freedom SOLO pericardial aortic valve. Eur J Cardiothorac Surg 2016;49:493–9

18. Zhigalov K, Sá MPBO, Gogia I, Chaduneli O, Adjailia EB, Mashhour A, Easo J, Wendt D, Ruhparwar A, Dapunt O, Weymann A, Eichstaedt HC. Outcomes and hemodynamics of Enable bioprosthesis in 432 patients: an afterword. Minim Invasive Ther Allied Technol. 2022 Feb;31(2):291-296.

19. Hernandez-Vaquero D, Vigil-Escalera C, Persia Y, Morales C, Pascual I, Domínguez-Rodríguez A, et al. Perceval or Trifecta to Prevent Patient–Prosthesis Mismatch. J Clin Med 2020;9:2964.

20. Sabik JF 3rd, Rao V, Lange R, Kappetein AP, Dagenais F, Labrousse L, Bapat V, Moront M, Weissman NJ, Patel HJ, Reardon MJ, Asch FM, Zeng C, Klautz RJM; PERIGON Investigators. One-year outcomes associated with a novel stented bovine pericardial aortic bioprosthesis. J Thorac Cardiovasc Surg. 2018 Oct;156(4):1368-1377.e5.

21. Barnhart GR, Accola KD, Grossi EA, Woo YJ, Mumtaz MA, Sabik JF, et al. TRANSFORM (Multicenter Experience With Rapid Deployment Edwards INTUITY Valve System for Aortic Valve Replacement) US clinical trial: Performance of a rapid deployment aortic valve. J Thorac Cardiovasc Surg 2017;153:241-251.e2.

22. Bavaria JE, Griffith B, Heimansohn DA, Rozanski J, Johnston DR, Bartus K, Girardi LN, Beaver T, Takayama H, Mumtaz MA, Rosengart TK, Starnes V, Timek TA, Boateng P, Ryan W, Cornwell LD, Blackstone EH, Borger MA, Pibarot P, Thourani VH, Svensson LG, Puskas JD; COMMENCE Trial Investigators. Five-year Outcomes of the COMMENCE Trial Investigating Aortic Valve Replacement With RESILIA Tissue. Ann Thorac Surg. 2023 Jun;115(6):1429-1436.
